# Toward Effective CO_2_ Reduction in an Acid Medium: Electrocatalysis at Cu_2_O-Derived Polycrystalline Cu Sites Immobilized within the Network of WO_3_ Nanowires

**DOI:** 10.1021/acsmeasuresciau.2c00010

**Published:** 2022-06-28

**Authors:** Iwona A. Rutkowska, Anna Chmielnicka, Maciej Krzywiecki, Pawel J. Kulesza

**Affiliations:** † Faculty of Chemistry, University of Warsaw, Pasteura 1, Warsaw PL-02-093, Poland; ‡ Institute of Physics−CSE, Silesian University of Technology, Konarskiego 22B, Gliwice PL-44-100, Poland

**Keywords:** carbon dioxide conversion, electrochemistry, acid medium, catalysis, hydrogen evolution tolerance, copper (I) oxide, hexagonal tungsten (VI) oxide nanowires, charge trapping and copper dispersion

## Abstract

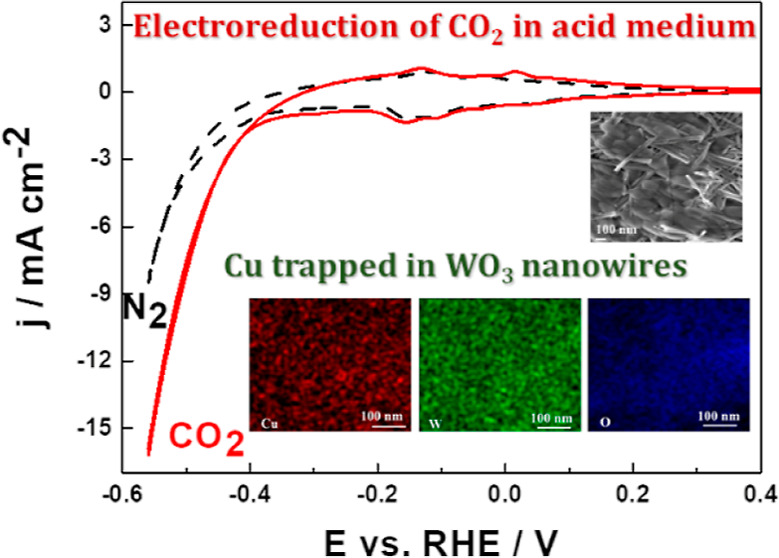

A hybrid catalytic system composed of copper (I)-oxide-derived copper nanocenters immobilized within the network of tungsten oxide nanowires has exhibited electrocatalytic activity toward CO_2_ reduction in an acid medium (0.5 mol dm^–3^ H_2_SO_4_). The catalytic system facilitates conversion of CO_2_ to methanol and is fairly selective with respect to the competing hydrogen evolution. The preparative procedure has involved voltammetric electroreduction of Cu_2_O toward the formation and immobilization of catalytic Cu sites within the hexagonal structures of WO_3_ nanowires which are simultaneously partially reduced to mixed-valence hydrogen tungsten (VI, V) oxide bronzes, H_*x*_WO_3_, coexisting with sub-stoichiometric tungsten (VI, IV) oxides, WO_3–*y*_. After the initial loss of Cu through its dissolution to Cu^2+^ during positive potential scanning up to 1 V (vs RHE), the remaining copper is not electroactive and seems to be trapped within in the network of hexagonal WO_3_. Using the ultramicroelectrode-based probe, evidence has also been provided that partially reduced nonstoichiometric tungsten oxides induce reduction of CO_2_ to the CO-type reaction intermediates. The chronocoulometric data are consistent with the view that existence of copper sites dispersed in WO_3_ improves electron transfers and charge propagation within the hybrid catalytic layer. The enhanced tolerance of the catalyst to the competitive hydrogen evolution during CO_2_R should be explained in terms of the ability of H_*x*_WO_3_ to consume protons and absorb hydrogen as well as to shift the proton discharge at Cu toward more negative potentials. However, the capacity of WO_3_ to interact with catalytic copper and to adsorb CO-type reaction intermediates is expected to facilitate removal of the poisoning CO-type adsorbates from Cu sites.

## 1 Introduction

The continuously rising levels of carbon dioxide (CO_2_), a greenhouse gas released through burning of fossil fuels, is a critical environmental issue and has triggered serious concerns and attention toward capture and conversion of the excessive amounts of CO_2_ in the atmosphere.^1−7^ Utilization of renewable energy sources to convert CO_2_ into simple organic fuels and utility chemicals constitutes an attractive strategy to mitigate the problem of excessive emissions of CO_2_ as well as permits to address the issues of the low-environmental-impact formation of energy-dense liquids that are convenient for storage and transportation.^8−10^ Different approaches, which include thermocatalytic, photocatalytic, biochemical, and electrocatalytic procedures,^2−7^ have been considered for the direct CO_2_-conversion processes. In this respect, the electrochemical CO_2_-reduction (CO_2_R) approach is one of the most attractive routes to produce high-energy-density fuels and valuable chemicals, such as carbon monoxide, formic acid, simple hydrocarbons, and alcohols (in addition to hydrogen), in a controllable and eco-friendly manner in aqueous or water-containing media under ambient conditions.^11−14^ Nevertheless, the activity, selectivity, and overall efficiency of electrochemical CO_2_R processes are still far from the practical needs for industrial applications. In this respect, there is a need for a better understanding of the reaction mechanisms and conditions determining the systems’ selectivity toward desired products.

Historically, both heterogeneous and homogeneous catalytic systems were considered for electrochemical CO_2_R conversions.^14−31^ Among catalytic materials, such metals as Cu, Au, Ag, Bi, Pb, or Pd; bimetallic (e.g., PdRu, CuAu, or AuAg) nanoparticles; certain nanostructured metal oxides (e.g., Cu_2_O, TiO_2_, ZnO, and ZrO_2_); functionalized carbon metalorganic complexes; and coordination compounds of transition metals or nitrogen-containing ligands, such as pyridine, bipyridine, and benzoimidazole, should be mentioned here. In particular, copper and its derivatives have exhibited unique properties permitting reduction of carbon dioxide to a series of hydrocarbons and C_*x*_H_*y*_O_*z*_-type oxo-hydro-carbons (e.g., alcohols, aldehydes) with reasonable Faradaic efficiencies.^12,32−34^ It is noteworthy that CO_2_R at Cu-based catalysts proceeds according to mechanisms involving typically more than two electrons per CO_2_ molecule, thus yielding products further reduced than carbon monoxide or formate.^5,28,33,35^ Nevertheless, the Cu-containing electrocatalysts require significant overpotentials, and selectivity remains an issue, particularly, when it comes to the formation the more reduced products. On mechanistic grounds, CO_2_ is first reduced to CO or HCOO^–^ through two-electron processes, where CO is believed to act as the reaction intermediate to be subsequently reduced, whereas HCOO^–^ appears to be a terminal product.^36,37^ In general, the selectivity of the reaction depends on the affinity of catalytic centers to adsorb the carbon monoxide intermediate,^38^ including its protonation or hydrogenation. The surfaces of Cu catalysts derived from copper oxides are covered with −OH species and favor the formation of oxygenated products (e.g., methanol) at relatively low overpotentials.^4,5,39,40^ For example, the emergence of the −CH_2_OH intermediate on the catalytic surfaces has been reported to be a key factor for a high methanol selectivity.^4,41^ It is generally accepted that copper-containing catalysts are the best available systems for direct CO_2_R to alcohols and hydrocarbons.^14,27,36,42^ Recent studies also imply that nanostructured copper catalysts in the form of nanoparticles,^43−48^ nanocubes,^49,50^ nanofoams,^51^ and nanowires^52^ are usually characterized by higher CO_2_R-current densities and better selectivity, in comparison to the performance of the systems of conventional sizes.^53,54^

As already mentioned, Cu nanostructures generated upon reduction of copper oxides, particularly Cu_2_O, show an improved CO_2_R efficiency at lower overpotentials.^54−57^ In this respect, the unique surface active centers have been correlated to grain boundaries existing in the oxide-derived Cu sites at the electrocatalytic interface.^40,54,58,59^ Among other important issues are the catalyst nanostructuring, an increase of the electrochemically active surface area, and a possibility of the appearance of low-coordinated atoms as active sites following the oxide reduction.^39,43,60−63^

Application of copper catalysts is also limited by hydrogen evolution side reaction through proton discharge or water reduction.^64−66^ To control the hydrogen evolution reaction as the competitive side process, the use of the proton-depleted electrochemical interfaces has been postulated. However, the CO_2_-electroreduction stoichiometric reactions do involve protons;^67^ consequently, a sufficient population of H_3_O^+^ ions or H atoms capable of interacting with the CO_2_-reduction adsorbates (reaction intermediates)^68^ is needed at the electrocatalytic interface. Perhaps the optimum catalyst would be hybrid in nature: it should generate active sites for adsorption (activation) of CO_2_ and suppress somewhat hydrogen evolution.

The feasibility of application of acid electrolytes is also tempting because CO_2_R in acidic media offers an avenue to eliminate almost completely the carbonate formation and crossover.^69,70^ Indeed, in alkaline and neutral media, where H_2_O is the proton source, a large fraction of CO_2_, rather than being reduced, reacts with OH^–^ to produce CO_3_^2–^, thus resulting in the decrease of the CO_2_R reaction efficiency.

In the present work, to circumvent the kinetically more favorable hydrogen evolution in acids, we propose a hybrid Cu-based catalyst in which unique copper active sites are intentionally stabilized and modified with the appropriate metal oxide nanostructures. Here, we consider Cu_2_O nanoparticles as a source of the oxide-derived copper catalytic element.^22,47,71,72^ Having in mind our recent electrocatalytic results with polytungstate-network-stabilized copper oxo assemblies^73^ and regarding successful utilization of the Cu_2_O films over-coated with WO_3_ nanowires for both electrochemical and photoelectrochemical reduction of carbon dioxide in near-neutral media, we have pursued research along this line and propose here a hybrid catalytic system composed of copper (I) oxide and tungsten (VI) oxide nanostructures exhibiting improved high selectivity toward CO_2_R relative to the competitive hydrogen evolution in an acid medium (0.5 mol dm^–3^ H_2_SO_4_). Upon reduction of WO_3_ in acids, hydrogen tungsten (VI, V) oxide bronzes (H_*x*_WO_3_, 0 < *x* < 1) and, at more negative potentials, sub-stoichiometric oxygen-deficient tungsten (VI, IV) oxides (WO_3–*y*_, 0 < *y* < 1) are formed.^67,74−80^ These partially reduced tungsten oxides are capable of sorption of hydrogen rather than inducing hydrogen evolution in the acid medium. By combining the copper and tungsten oxide reactivities, we develop the system that is intrinsically more active toward CO_2_R than toward hydrogen evolution. In other words, we demonstrate that one can pursue the CO_2_ conversion in the acidic medium (barely explored so far)^69,70^ under low-temperature conditions. The appearance of both hydrogen (within tungsten oxide) and CO or simple C_*x*_H_*y*_O_*z*_ oxo-hydro-carbon intermediates (on copper sites) at the electrocatalytic interface should, in principle, permit us to pursue the electrochemically driven Fischer–Tropsch-type syntheses (alcohols).

## 2 Experimental Section

### 2.1 Materials and Preparative Procedures

Chemical reagents were analytical grade materials. Tungsten (VI) oxide nanowires, copper (I) oxide nanopowder, and 5% Nafion-1100 solution were purchased from Aldrich. Sulfuric acid was from POCh (Gliwice, Poland).

All solutions were prepared using doubly distilled and subsequently deionized (Millipore Milli-Q) water. They were deoxygenated by bubbling with high-purity nitrogen for at least 30 min. Unless otherwise stated, before the electrochemical diagnostic measurements were performed, electrolytes were saturated with CO_2_ of premium quality (from Air Products) for at least 30 min, and the CO_2_-saturated atmosphere was kept over the investigated solutions during the actual experiments. Measurements were carried out at room temperature (22 ± 2 °C).

Suspension (ink) of copper (I) oxide nanoparticles was prepared by dispersing 1.0 mg of Cu_2_O nanopowder in 0.1 cm^3^ of deionized water using an ultrasonic bath for 2 h. Later, 2.0 μdm^3^ of the ink was placed onto the electrode surface to yield the cooper (I) oxide loading of 300 μg cm^–2^. The tungsten oxide suspension was fabricated in an analogous manner but by dispersing 1.0 mg of WO_3_ nanowires in 0.1 cm^3^ of deionized water. Later, 2.0 μdm^3^ of an aqueous suspension of WO_3_ nanowires was introduced to form an outer layer on the Cu_2_O film. The loading of WO_3_ nanowires was equal to 300 μg cm^–2^. As a rule, the bilayer Cu/W-oxide catalytic films were over-coated and stabilized with ultrathin layers of the Nafion polyelectrolyte fabricated by depositing 1 μL of the Nafion solution (prepared by admixing 5% mass of the commercial Nafion dispersion in ethanol with water at a 1 to 10 volumetric ratio).

Before the representative electrocatalytic measurements were performed, the electrodes were conditioned by voltammetric potential cycling (5 full potential cycles at 10 mV s^–1^; quiet time, 20 s at the starting potential) in the potential range from 0.40 to −0.56 V [vs reversible hydrogen electrode (RHE)] in the CO_2_-saturated 0.5 mol dm^–3^ H_2_SO_4_.

### 2.2 Electrochemical Characterization

All electrochemical measurements were performed using a CH Instruments (Austin, TX, USA) Model 760D workstation in the three-electrode configuration. The glassy carbon working electrode was utilized in a form of a disk of a geometric area of 0.071 cm^2^. The reference electrode was the K_2_SO_4_-saturated Hg_2_SO_4_ electrode; it was separated from the investigated solutions by inserting into the Luggin tube and placed close to the working electrode. Carbon rod was used as the counter electrode. All potentials were reported, recalculated, and expressed versus RHE. Prior to experiments, the working electrode was polished with aqueous alumina slurries (grain size, 0.05 μm) on a Buehler polishing cloth.

### 2.3 Microscopic, Structural, and Spectroscopic Characterization

Transmission electron microscopy (TEM) experiments were carried out with a Libra 120 EFTEM (Carl Zeiss) operating at 120 kV. Scanning electron microscopy (SEM) measurements and energy-dispersive X-ray (EDX) analysis were achieved using a MERLIN FE-SEM (Carl Zeiss) equipped with an EDX analyzer (Bruker). The energy-dispersive spectroscopy (EDS) system together with an XFlash Detector 5010 125 eV Quantax Bruker microscope (15.0 kV acceleration voltage and 10 K magnification) was used to obtain the elemental analysis information.

Infrared spectra were measured with a Shimadzu 8400 FTIR spectrometer. The infrared reflectance absorption spectra of tungsten oxide deposits on gold-covered glass were recorded using a Specular Reflectance Accessory Model 500 provided by Spectra Tech. The beam incidence angle was equal to 80° with respect to the surface normal. Typically, 1000 scans were averaged for a single reflectance spectrum.

X-ray diffraction (XRD) patterns were obtained on a X’Pert Pro Panalytical diffractometer with Co Kα radiation (*k* = 1.78901 Å). The XRD measurements were carried out by a step scanning method (2 h range; from 3 to 60°), the scanning rate was 0.03°/s, and the step time was 3 s. X-ray photoelectron spectroscopy (XPS) investigations were carried out using a multi-chamber ultra-high vacuum experimental setup (base pressure 5 × 10^–8^ Pa) equipped with a PREVAC EA15 hemispherical electron energy analyzer equipped with a 2D-MCP detector. The samples were irradiated with an Al Kα X-ray source (PREVAC dual-anode XR-40B source, energy 1486.60 eV). For the survey spectra, the pass energy was set to 200 eV (with scanning step 0.9 eV). Particular energy regions to 100 eV were achieved with a scanning step of 0.05 eV. The binding energy (BE) scale of the analyzer was calibrated to Au 4f_7/2_ (84.0 eV). Recorded data were fitted utilizing CASA XPS embedded algorithms and relative sensitivity factors. For background subtraction, the Shirley function was used. The components were fitted with a sum of Gaussian (70%) and Lorentzian (30%) lines. For the peaks in the same BE region, the full width at half-maximum values were allowed to vary within a narrow range. The estimated uncertainty for components’ energy position determination was 0.1 eV.

The CO_2_R products were identified using gas chromatography coupled with mass spectrometry (GC-MS) as well as by nuclear magnetic resonance (NMR). Volatile (gaseous) CO_2_R products were analyzed using gas chromatography (Agilent Technologies 7890A GC System) equipped with a silica-based Stationary Phase GS-GASPRO column, thermal conductivity detector, and flame ionization detector. Helium was utilized as a carrier gas, and sulfur hexafluoride (SF_6_) was used as an internal standard. After 2 h of electrolysis, 30 μL of the gas sample [collected with a GC injector from the gas collector] was mixed with 10 μL of SF_6_ and, subsequently, injected to the inlet of the gas chromatograph. The gas collector was in the form of an upturn funnel with the sept tip submerged in the electrolyte and placed directly over the working electrode inserted at the bottom of the electrochemical cell. To confirm the formation of liquid volatile products such as methanol, during CO_2_ reduction, the head space sampling (for GC-MS determination) involved heating the shut vial at 75–80 °C in a water bath for 45 min. The NMR diagnostic experiments were performed using a Bruker AV300 spectrometer. Chemical shifts (δ) are given in ppm using D_2_O as a solvent. The ^1^H chemical shifts were measured relative to solvent peaks considering TMS = 0 ppm.

## 3 Results and Discussion

### 3.1 Physicochemical Identity and Electrocatalytic Activity of Cu_2_O and WO_3_ in the Acid Medium

First, nanostructured forms of Cu_2_O nanoparticles and WO_3_ nanowires have been characterized as separate catalytic components. The XRD patterns of these materials are illustrated in the Supporting Information (Figures S1 and S2). The appearance of well-defined diffraction peaks positioned at 36.4, 42.5, 61.4, and 73.4° (Figure S1) should be correlated with (111), (200), (220), and (311) crystalline planes of the cubic copper (I)-oxide phase (JCDS 78-2076), respectively.^81^ In the present work, Cu_2_O nanoparticles serve as the starting oxide-derived source of active copper catalytic sites following reduction.^39,40,43,54,58−63^ The XRD pattern presented in Figure S2 is consistent with the predominantly hexagonal tungsten (VI) oxide hydrate characterized by a tunnel structure with zeolitic water molecules existing along the tunnels.^82^ Indeed, the XRD pattern of WO_3_ nanowires shows a sharp peak located at 28.2° corresponding to the (200) plane of hexagonal WO_3_ (Joint Committee on Powder Diffraction Standards, JCPDS #33-1387). Two additional peaks at 14.0 and 24.3° should be attributed to the diffraction from (100) and (110) planes of WO_3_, respectively. It was reported for hexagonal WO_3_ that the system’s stability and proton mobility, which were required to provide charge balance during redox transitions,^74,75^ were promoted by the existence of water chains inside the structural channels of hydrous hexagonal WO_3_.^83^ Thus, the high surface area created from open and rigid tunnels (diameter 367 pm) in hexagonal WO_3_ not only favors applications in electrochemical energy storage and selective gas adsorption (e.g., CO_2_) but also permits selective ion transfers and makes insertion of hydrated copper ions (diameters below 160 pm)^84^ possible. Finally, the adsorption of CO (CO_2_R intermediate) on WO-terminated and, particularly, O-terminated (chemisorption) surfaces of hexagonal WO_3_ (001) surfaces was postulated.

**Figure 1:**
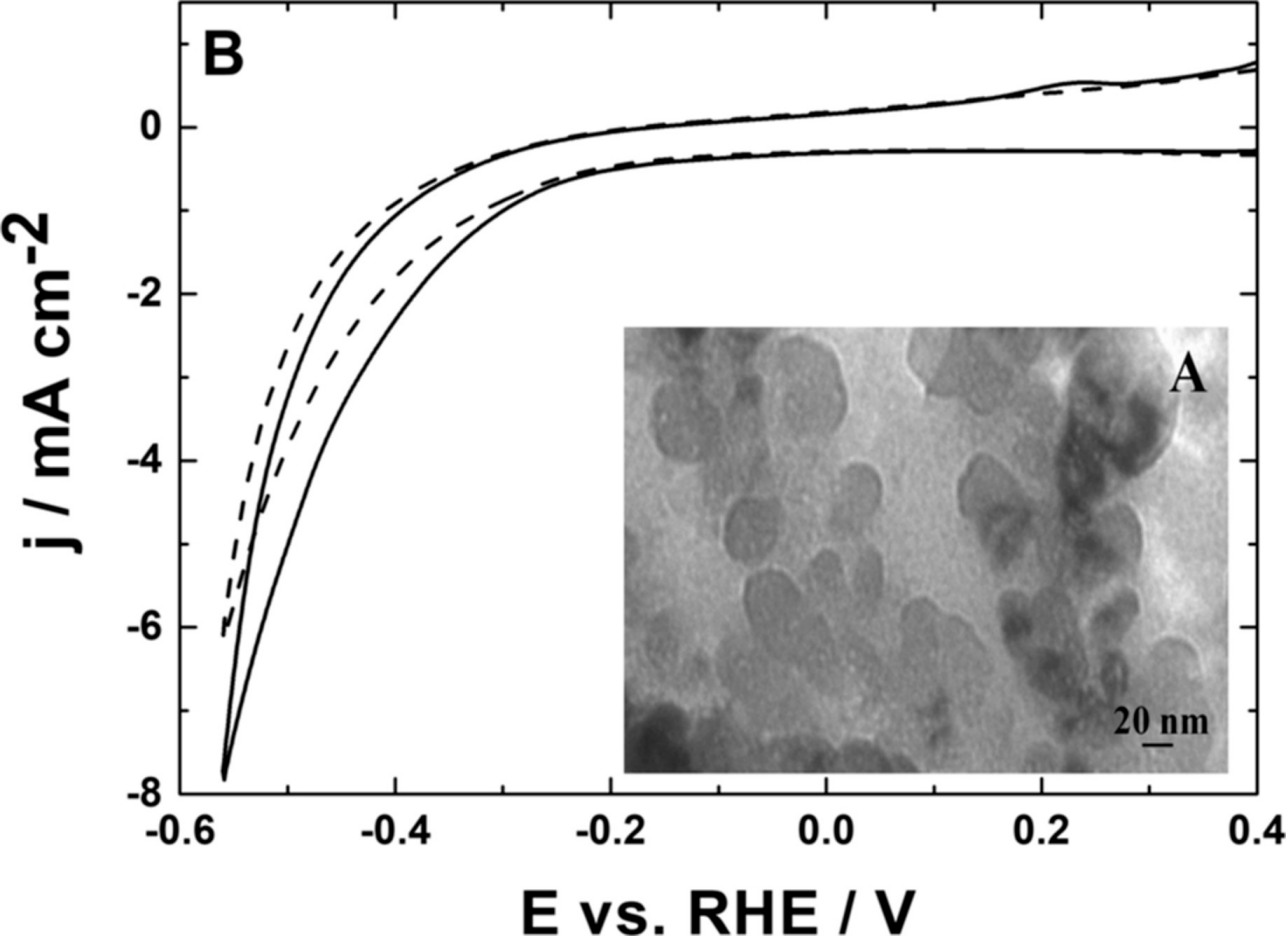
(A) Transmission electron micrographs of Cu_2_O nanostructures. (B) Cyclic voltammetry of Cu_2_O nanoparticles (deposited on the glassy carbon disk) in the absence (dashed line) and presence (solid line) of carbon dioxide (saturated solution). Electrolyte, 0.5 mol dm^–3^ H_2_SO_4_. Scan rate, 10 mV s^–1^.

**Figure 2:**
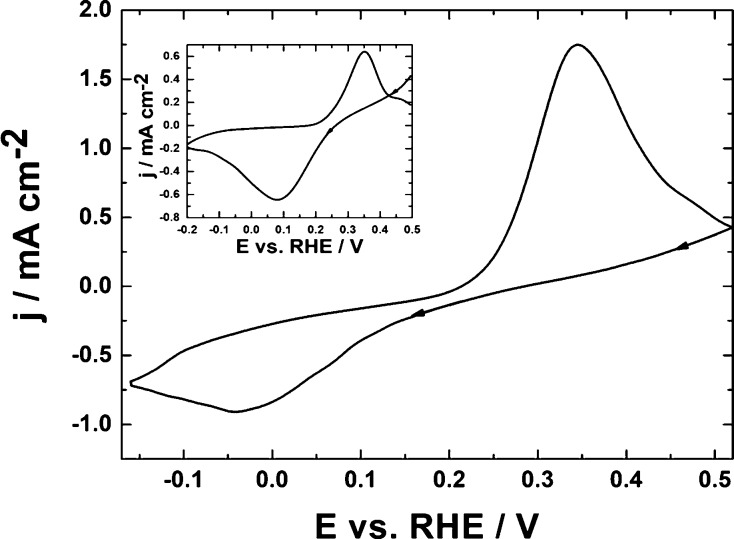
Stripping-type voltammograms recorded following adsorption of CO_2_ at Cu_2_O nanoparticles (deposited on the glassy carbon disk). Inset: cyclic voltammetry of Cu_2_O nanoparticles. Electrolyte, 0.5 mol dm^–3^ H_2_SO_4_. Scan rate, 10 mV s^–1^.

Figure 1A (inset) illustrates the TEM image of Cu_2_O nanoparticles which have been used here for the preparation of catalytic inks and, later, for the formation of catalytic layers (upon deposition) on glassy carbon substrates. The nanostructures seem to be spherical (with diameters ranging from 20 to 40 nm), but they tend to coalesce to form agglomerates of sub-micrometer sizes. To get insight into the basic electrochemical and electrocatalytic properties of Cu species resulting from the reduction of Cu_2_O films, the oxide particles were first deposited on glassy carbon and subjected to voltammetric examination in deoxygenated 0.5 mol dm^–3^ H_2_SO_4_. Figure 1B illustrates the voltammetric responses recorded in the presence (solid line) and absence (dashed line) of carbon dioxide. In both cases, the voltammetric scans have been initiated (toward negative potentials) from the moderately positive potential of 0.4 V (vs RHE), where Cu_2_O still exists but is not oxidized to CuO.^85^ To avoid excessive hydrogen evolution or the compound’s degradation, care has been exercised to apply neither too negative nor too positive potentials. At potentials higher than 0.5 V (inset of Figure 2), the oxidation of Cu_2_O to Cu(II) species takes place. The actual voltammetric peak appearing in the range from 0.5 to 0.8 V has shown some degree of overlapping most likely due to the formation of both copper (II) ions and copper (II) oxide species (followed by their dissolution). Furthermore, generation of metallic copper (through electroreduction of copper ions mentioned above) tended to proceed in 0.5 mol dm^–3^ H_2_SO_4_ according to the one-step mechanism (single peak around 0.45 V in Figure 2, inset), rather than via the two-step reaction involving the formation of the Cu(I) forms. However, upon application of potentials lower than −0.2 V (vs RHE), where copper certainly existed in the form of metallic (Cu^0^) sites, the hydrogen evolution reaction was operative (dashed line in Figure 1B).

Unlike the typical behavior of CO_2_ at metallic copper electrodes in near-neutral media^85^ where the CO_2_R currents tend to decrease (relative to the competitive hydrogen evolution) due to the appearance of inhibiting CO-type adsorbates, the present results imply a somewhat different mechanism related to the CO_2_ electroreduction at copper sites originating from Cu_2_O in 0.5 mol dm^–3^ H_2_SO_4_. It is apparent from the data of Figure 1B (compare dashed and solid lines) that electroreduction of CO_2_ starts at ca. −0.2 V (vs RHE) and continues at lower potentials, and it is accompanied by hydrogen evolution. Electroreduction of carbon dioxide is known to proceed with the formation of the CO-poisoning intermediate (adsorbate),^27,36,47,66,73^ but the CO-poisoning effect has not been so severe in the present case. Obviously, the observed redox currents (Figure 1B, solid line) originate from the proton-induced CO_2_R processes and the competing proton-discharge (hydrogen evolution) reaction. In addition to hydrogen, the reduction products are likely to include (but they are not limited to) carbon monoxide, formate, and alcohols.^14,27,35,36,47,66^ Hydrocarbons (e.g., CH_4_ and C_2_H_4_) are not expected to be generated in the investigated range of potentials.

Adsorptive interactions between copper oxo species and CO_2_ can be envisioned. Careful examination of the voltammetric characteristics of CO_2_ at the Cu_2_O nanoparticles in the positive potential range (where CO_2_ is not expected to be electroreduced) provides some evidence in this respect. It is apparent from Figure 2 that following conditioning the Cu_2_O deposit at 0.5 V for 20 s in the presence of CO_2_, carbon dioxide has apparently undergone adsorption on the surfaces of copper oxo species and has stabilized the system to permit its voltammetric characterization. The development of the well-defined surface-type anodic stripping peak (at about 0.4 V, most likely involving the oxidation of the CO-type adsorbate to CO_2_) during positive potential scanning from ca. −0.15 to 0.5 V should be noted here. Such interfacial characteristics (involving most likely the CO_2_-adsorptive reduction to CO, followed by the oxidative stripping) were previously demonstrated for noble metal systems.^13,25,54,85,86^ The present observation is consistent with the existence of specific adsorptive interactions between copper oxo species and CO_2_ in the acid medium.

**Figure 3:**
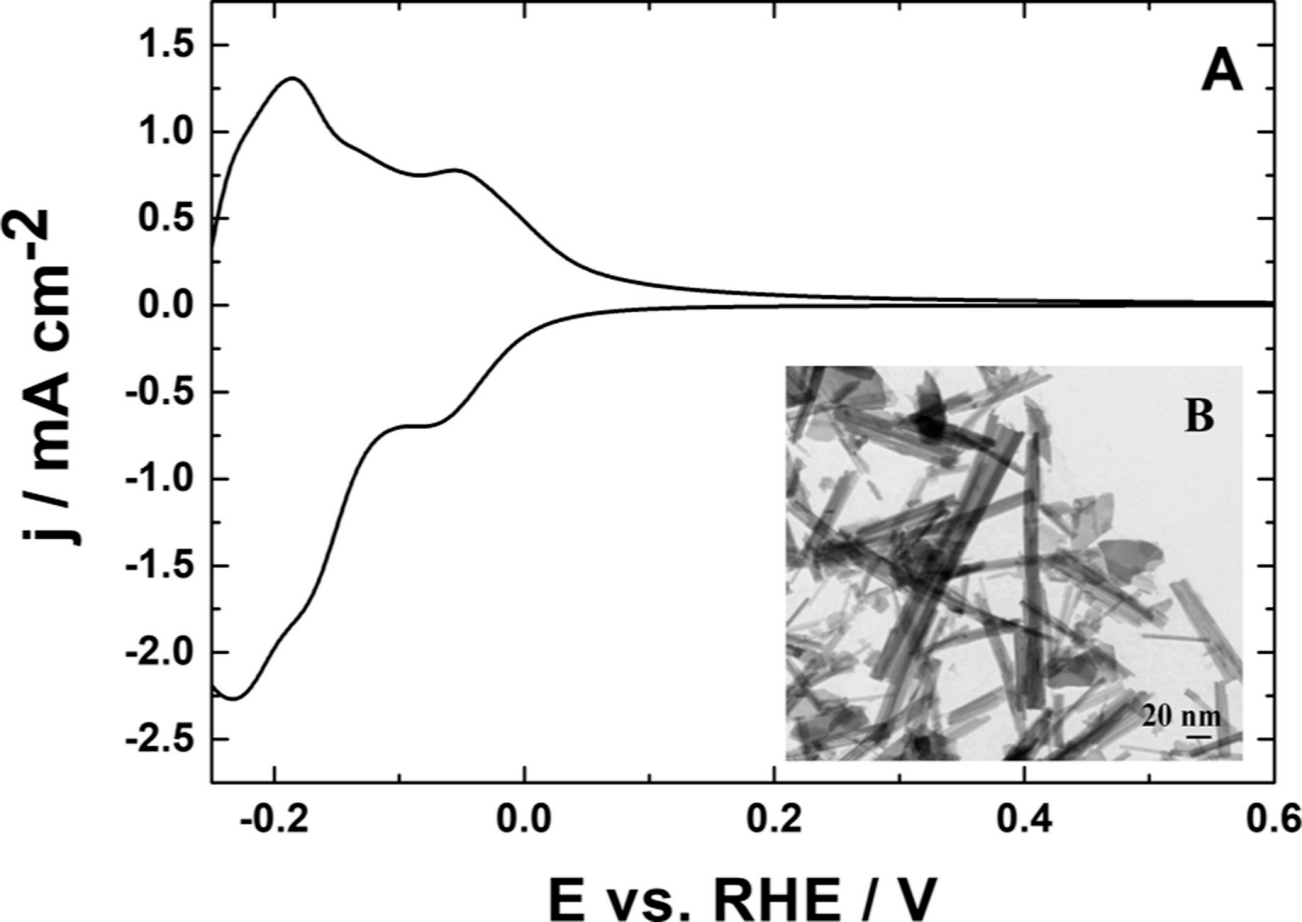
(A) Cyclic voltammetry of WO_3_ nanowires deposited on glassy carbon. Electrolyte, 0.5 mol dm^–3^ H_2_SO_4_. Scan rate, 10 mV s^–1^. (B) Transmission electron micrograph of WO_3_ nanowires.

Tungsten oxides are characterized by fast redox transitions in acid media, and in addition to well-known electric, electrochromic, or photoelectrochemical properties, they exhibit—upon partial reduction—high catalytic activity during reductions of electrochemically inert reactants.^67,74,75^Figure 3A illustrates cyclic voltammetric behavior (in 0.5 mol dm^–3^ H_2_SO_4_) of WO_3_ nanowires deposited on glassy carbon. Judging from the transmission electron micrograph of WO_3_ material (Figure 3B, inset), the nanowires appear as “needles” having diameters on the level of 10–20 nm, but their lengths are in the submicrometer range (reaching even 350 nm). Two dominating sets of the system’s voltammetric peaks (Figure 3A) appearing at potentials lower than 0.3 V reflect redox transitions of tungsten oxide consistent with the reversible formation of two partially reduced forms of WO_3_, namely, hydrogen tungsten oxide bronzes of the type H_*x*1_WO_3_ and H_*x*2_WO_3_.^67,74−80^ Both forms are mixed-valent and contain W^VI^ and W^V^ ionic sites, but they differ in the degree of reduction and nonstoichiometry.^75^ The voltammetric pattern is analogous to that reported elsewhere, illustrating the performance of hexagonal tungsten oxide hydrate.^82^ On the whole, the electrochemical behavior of the system is characterized in the acid medium by fast and reversible redox transitions. The fact that the pair of peaks at about −0.2 V is not exactly symmetrical around the zero current axis and the reduction peak current appears relatively higher than the respective oxidation one reflects most likely contribution from the parallel formation of lower tungsten oxides, WO_3–*y*_ (where W^VI^ and W^IV^ ionic sites coexist),^75^ accompanied by consumption of protons and sorption of hydrogen.^67,74,75,87^ As a consequence of the latter phenomenon, it was found that hydrogen evolution tended to be somewhat suppressed at WO_3_-modified electrodes relative to the performance of bare electrode substrates.^74,75,87^ Finally, because the experiments are performed in acidic solutions (Figure 3A), rather than neutral media,^58^ the redox transitions are proton-supported, and thus, the respective peaks are well-defined and reversible.

**Figure 4:**
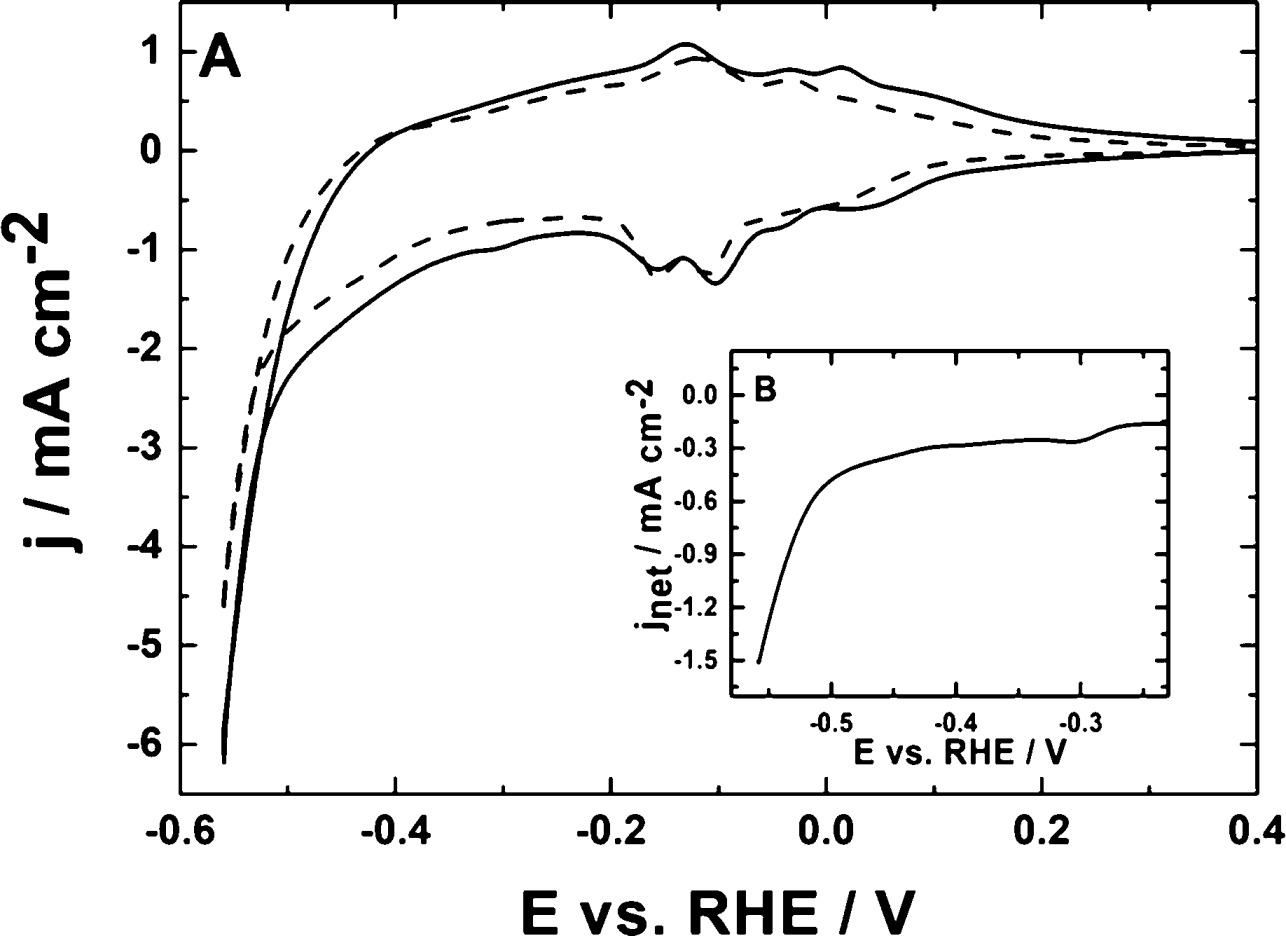
(A) Cyclic voltammetric responses of WO_3_ nanowires (deposited on glassy carbon) recorded in the absence (dashed line) and presence (solid line) of carbon dioxide (saturated solution). (B) Inset: background-subtracted forward voltammetric responses for the reduction of CO_2_. Electrolyte, 0.5 mol dm^–3^ H_2_SO_4_. Scan rate, 10 mV s^–1^.

Additional attention was paid to the comparison of electrochemical responses of WO_3_ films (on glassy carbon) in the presence (CO_2_-saturated in 0.5 mol dm^–3^ H_2_SO_4_) and absence of carbon dioxide (Figure 4A). The initial scan (toward negative potentials) was started from 0.4 V (vs RHE) where WO_3_ was fully oxidized (see Figure 3A). At potentials lower than 0.2 V (vs RHE), the reduction of WO_3_ to the already mentioned nonstoichiometric tungsten oxides of the type H_*x*_WO_3_ and WO_3–*y*_ became operative. At potentials more negative than −0.2 V, the formation of the oxygen-deficient lower tungsten oxides of the WO_3–*y*_ type (Figure 4A, dashed line) appeared to induce reduction of CO_2_ (Figure 4A, solid line). The reduction of CO_2_ was even more evident from the background-subtracted voltammetric response (Figure 4B, inset): while CO_2_R started to be evident at −0.2 V, the process was accompanied by hydrogen evolution below −0.5 V. Careful examination of voltammetric responses in the presence and absence of CO_2_ (Figure 4A) implies some differences in voltammograms in the potential range from 0.15 to −0.25 V, where redox transitions of hydrogen-rich tungsten bronzes (H_*x*_WO_3_) are operative. These changes are likely to be induced by interfacial interactions between CO_2_ and H_*x*_WO_3_.

It is apparent upon comparison of the data of Figures 1B and 4A (dashed lines recorded in the absence of CO_2_) that the onset of hydrogen evolution appears at the more negative potential of −0.5 V during cathodic polarization at WO_3_ (Figure 4A) relative to the appearance of the hydrogen wave starting from −0.3 V at copper sites originating from Cu_2_O (Figure 1B). This observation can be explained in terms of the partial reduction of tungsten oxides accompanied by sorption of hydrogen^67,75^ proceeding before hydrogen evolution is effective.

### 3.2 Preparation, Characterization, and Performance of the Hybrid Cu/WO_3_ Film

**Figure 5:**
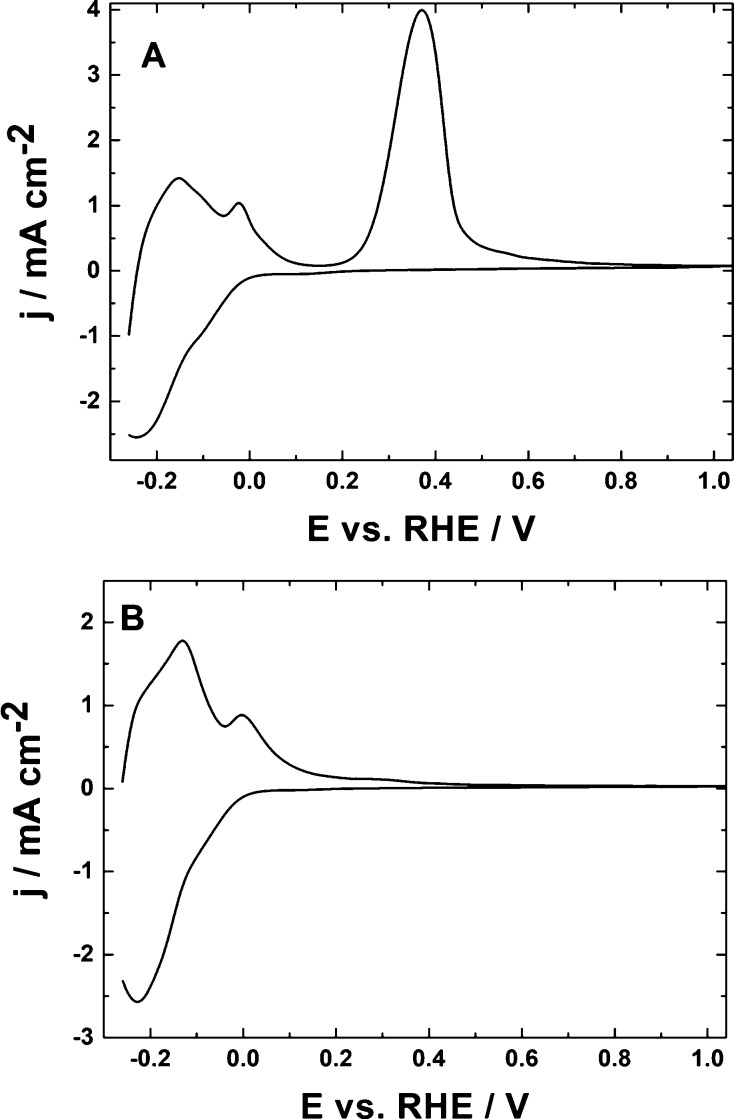
Cyclic voltammetric responses of the bilayer system of Cu_2_O over-coated with WO_3_ nanowires (deposited on glassy carbon) recorded during the (A) first cycle and (B) second cycle. Electrolyte, 0.5 mol dm^–3^ H_2_SO_4_. Scan rate, 10 mV s^–1^.

The hybrid film composed of copper sites (generated upon reduction of Cu_2_O nanoparticles) immobilized within the network of WO_3_ nanowires (Cu/WO_3_) has been prepared by sequential deposition, as described in the Experimental Section. SEM of the initially deposited bilayer film composed of Cu_2_O nanostructures over-coated with WO_3_ nanowires is illustrated in the Supporting Information as Figure S3. Later, the film has been subjected to conditioning by voltammetric cycling in the potential range from 1 to −0.25 V. Under such conditions, Cu(I) and Cu(II) ionic sites are expected to interact,^88^ form insoluble species,^89^ undergo reduction to Cu^0^, and get intercalated within tunnels existing in hexagonal WO_3_. It is apparent from Figure 5A that the excess of copper ions (unbound to WO_3_) is removed (stripped) from the electrode surface to solution in the initial positive-going scans. Indeed, the appearance of a fairly large anodic peak at about 0.4 V (Figure 5A) is consistent with the oxidation of unbound Cu species to Cu^2+^ ions, followed by their removal to solution. Later, despite potential excursions up to 1 V, steady-state (with no noticeable copper stripping) voltammetric responses have been obtained (Figure 5B). Consequently, as a rule, all electrocatalytic studies have been performed after subjecting the hybrid film to preconditioning involving at least six full potential cycles in the supporting electrolyte, that is, until the steady-state voltammogram has been obtained. EDS analytical measurements clearly imply the presence of copper in the hybrid Cu/WO_3_ film at the approximate atomic ratio of Cu to W equal to 1:7. The fact that the existing copper sites do not show any electroactivity allows to infer that first, no copper exists directly on the glassy carbon electrode surface and, second, the remaining copper sites are either strongly attached to the WO_3_ surfaces or have been incorporated into hexagonal WO_3_ structures, including the oxide tunnels. As already mentioned, even the radii of hydrated copper ions are smaller than the radius of the tunnel (367 pm).^83^

**Figure 6:**
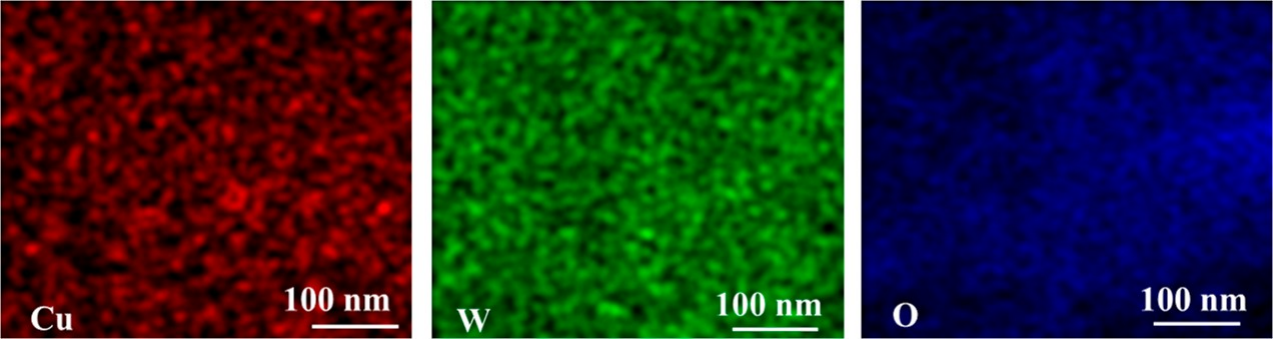
EDS mapping of the hybrid Cu/WO_3_ catalyst.

Simple comparison of data of Figures 3A and 5B implies some changes in the voltammetric characteristics of WO_3_ upon interacting with copper species coming from Cu_2_O. Judging from the fact that the WO_3_ first reduction peak at −0.07 V (Figure 3A) is largely suppressed in favor of the second reduction peak at ca. −0.22 V (Figure 5B), the copper component sites are present, indeed, in the hybrid film. The stability of the voltammetric response is consistent with the view that copper sites, while intercalated into the network of hexagonal WO_3_, are not stripped off into solution during voltammetric potential cycling. The presence and uniform distribution of both Cu and W components (in addition to O) in the hybrid Cu/WO_3_ catalytic films have been confirmed through EDS mapping (Figure 6).

Finally, it should be mentioned that the observed discrepancies between cathodic and anodic charges in the cyclic voltammetric responses of both Cu-free and Cu-containing WO_3_ films (Figures 3A and 5B) are characteristic of tungsten oxides in the acid medium,^74,75,77,78,87^ and they do not cease during prolonged potential cycling. The above feature reflects intrinsic proton discharge phenomena induced by electrons highly delocalized between W^VI^ and W^V^ ionic sites existing in nonstoichiometric tungsten oxide hydrogen bronzes.

**Figure 7:**
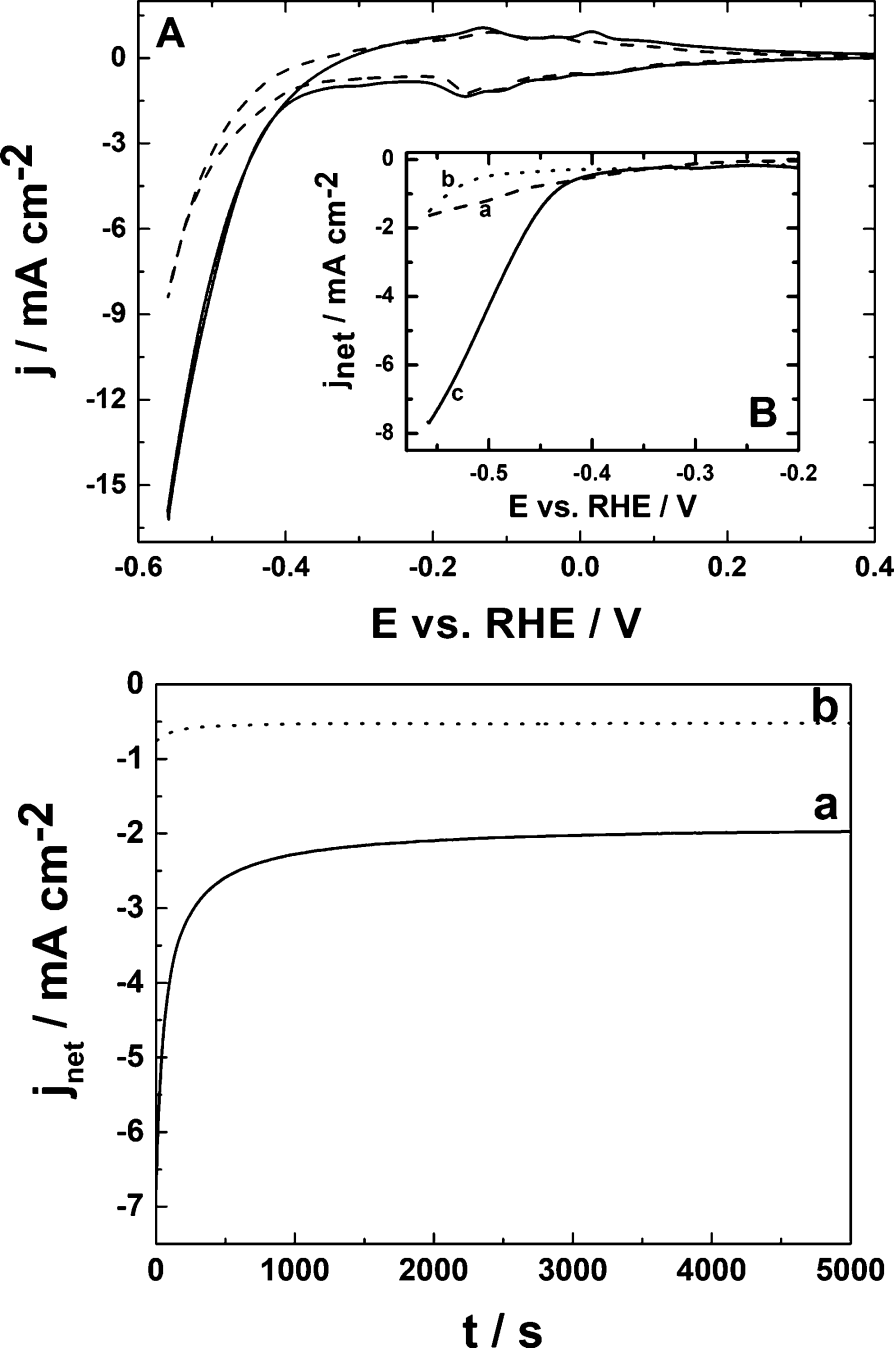
(A) Cyclic voltammetric responses recorded at 10 mV s^–1^ for the Cu/WO_3_ film (deposited on glassy carbon) in the absence (dashed line) and presence (solid line) of carbon dioxide (saturated solution). (B) Inset illustrates background-subtracted voltammetric responses for the reduction of CO_2_ at (a) Cu_2_O-derived Cu, (b) pristine WO_3_, and (c) Cu/WO_3_. (C) Chronoamperometric responses recorded upon application of −0.45 V during reduction of CO_2_ at (a) Cu/WO_3_ film (solid line) and (b) bare (Cu-free) WO_3_ (dotted line). Electrolyte, 0.5 mol dm^–3^ H_2_SO_4_.

Figure 7A illustrates voltammetric responses of the hybrid Cu/WO_3_ system recorded in both the presence (solid line) and absence (dashed line) of carbon dioxide. In comparison to the characteristics of copper sites generated from the pristine Cu_2_O film (Figure 1B), the dynamics of growth of the hydrogen evolution currents seems to be somewhat suppressed by the presence of sub-stoichiometric WO_3_ species (Figure 7A). It is also apparent, upon comparison of dashed lines in Figures 4A and 7A, that the performance of the Cu/WO_3_ system resembles the pattern observed for the WO_3_ component, rather than the behavior at pristine copper (Figure 1B, dashed line). However, in the case of Cu/WO_3_, CO_2_R currents are much higher, and they differ significantly from the background response recorded in the absence of CO_2_ (Figure 7A). Contrary to the behavior of CO_2_ at simple copper electrodes, where hydrogen evolution not only is competitive but also becomes the predominant reaction hiding the CO_2_ reduction (Figure 1B), the present result (Figure 7A) is consistent with fairly high electrocatalytic selectivity (with respect to hydrogen evolution) and appreciable activity of the hybrid Cu/WO_3_ system during CO_2_R reaction. It is commonly accepted that in strong acids, the efficiency for the formation of CO_2_R products is rather low.^70^ Here, it is evident from comparison (Figure 7B, inset) of background-subtracted (net) current densities at potentials lower than −0.4 V (vs RHE) that the Cu/WO_3_-catalyzed CO_2_ reduction currents (curve c) are significantly larger than those originating from the activities of single components, Cu_2_O-derived Cu (curve a) and WO_3_ (curve b), investigated under analogous conditions. Thus, the possibility of synergistic effects cannot be excluded.

Some attention has also been paid to the simultaneous evolution of hydrogen, which proceeds either through the reduction of protons (2H^+^ + 2e^–^ → H_2_) or through reduction of water molecules (2H_2_O + 2e^–^ → H_2_ + 2OH^–^). Here, the proton-discharge mechanism is obviously dominating because experiments have been performed in a strong acid. It was demonstrated before that CO_2_R suppresses hydrogen evolution originating from proton reduction.^66^ Here, among important issues is consumption of protons not only during CO_2_R but also during the formation of hydrogen bronzes (H_*x*_WO_3_). The surface roughness of the tungsten oxide component may also facilitate adsorption of both CO_2_ and CO as well as the related interfacial charge transfers. While at the pristine copper catalyst (Figure 1B), hydrogen evolution is the dominating reaction at potentials lower than −0.2 V, the formation of H_2_ at copper-free WO_3_ (Figure 4A) is relatively suppressed and shifted toward more negative potentials (−0.5 V). Consequently, the Cu-containing WO_3_ system becomes more selective favoring CO_2_R rather than the competitive hydrogen evolution. Indeed, the reduction currents observed at the hybrid Cu/WO_3_ are ca. twice larger in the presence of CO_2_ than in the absence of CO_2_. In other words, the hydrogen evolution reaction is suppressed (relative to the behavior of pristine Cu_2_O-derived Cu) and shifted in a manner resembling the behavior of copper-free WO_3_.

Among other important issues is persistence of the activity and stability of the background-subtracted steady-state electrocatalytic response of Cu/WO_3_ during CO_2_R under chronoamperometric conditions (Figure 7C, solid line a). This behavior is consistent with the fairly high activity of Cu/WO_3_, relative to the analogous performance of simple Cu-free WO_3_ (Figure 7C, dotted line b). The durability of the response implies good stability of the system as well as reasonably good resistance to poisoning through accumulation of CO-type intermediates at the electrocatalytic interface. On the contrary, current densities have tended to decrease in a case of the chronoamperometric monitoring of CO_2_R at simple Cu_2_O-derived Cu (Figure S4). It is likely that the copper sites have undergone either deactivation through poisoning or decomposition caused by relatively more pronounced hydrogen evolution.

### 3.3 Validation of Reaction Products

The gaseous CO_2_R products were subjected to gas chromatographic analysis (GC-MS). It was not surprising that in the acid medium and in the investigated range of potentials down to −0.6 V, hydrogen occurred to be the only gaseous CO_2_R product. No carbon monoxide was detected during chromatographic analysis. Obviously, at more negative potentials, the contribution from H_2_ evolution tended to increase. Indeed, upon application of potentials lower than −0.9 V, hydrogen was a dominating product, whereas methane (CH_4_) and other hydrocarbons (e.g., C_2_H_4_) were detected in trace amounts during the CO_2_ electroreduction (Figure S5). To avoid decomposition or reorganization of catalytic films, the potential excursion below −0.6 V was further not considered here.

The volatile liquid products, such as methanol, were identified using GC-MS following long-term (2 h) electrolysis (at −0.45 V) of CO_2_-saturated 0.5 mol dm^–3^ H_2_SO_4_ (deoxygenated) using the Cu/WO_3_-modified working electrode (glassy carbon) of 2 cm^2^ geometric surface area. The initial GC-MS determination was based on the head space sampling which necessitated heating the shut vial at 75–80 °C in a water bath for 45 min. The mass spectrum clearly indicated the presence of ion peaks at the 29, 31, and 32 *m*/*z* values which are typically attributed to methanol. For carbon monoxide detection, the *m*/*z* ratio equal to 12 was used, but no CO was found. Comparative measurements with standard solutions of methanol and ethanol were also performed. While methanol appeared as the main reaction product, there was no evidence for the formation of ethanol and other volatile reaction products. Additional chromatographic experiments were performed using GC-MS coupled with a JAS AED (Universal Injection System) atomic emission detector. The gaseous products were trapped in 50 mg molecular sieves (50–80 mesh) previously heated to 200 °C under vacuum for 12 h. Later, the volatile products were desorbed from the molecular sieves by heating the adsorbent to 200 °C without exposure to air, and the collected gas was analyzed using GC coupled with microwave-induced plasma excitation and AED with carbon 193 nm spectral line for recording chromatograms. Here, an HP-Innowax column was used for analyte separation. Methanol was detected as the only volatile product trapped following electrolysis (see GC-AED chromatogram in the Supporting Information as Figure S6). To deal with nonvolatile products, which concentrations were below limits of detection, the solution aliquots were preconcentrated by desiccation. Even then, neither formic acid nor acetic acid could be detected. In conclusion, methanol was detected as the main CO_2_R product. However, due to the limited trapping efficiency of gaseous products, the result could not be quantitative.

Finally, ^1^H NMR diagnostic measurements, involving addition of small amounts of the sample to the deuterated solvent (to avoid huge H_2_O-solvent absorption), were also conducted. There were two peaks, one for D_2_O and the other for CH_3_OH, in the NMR spectrum (Figure S7). There was no evidence for ethanol and formic acid.

**Figure 8:**
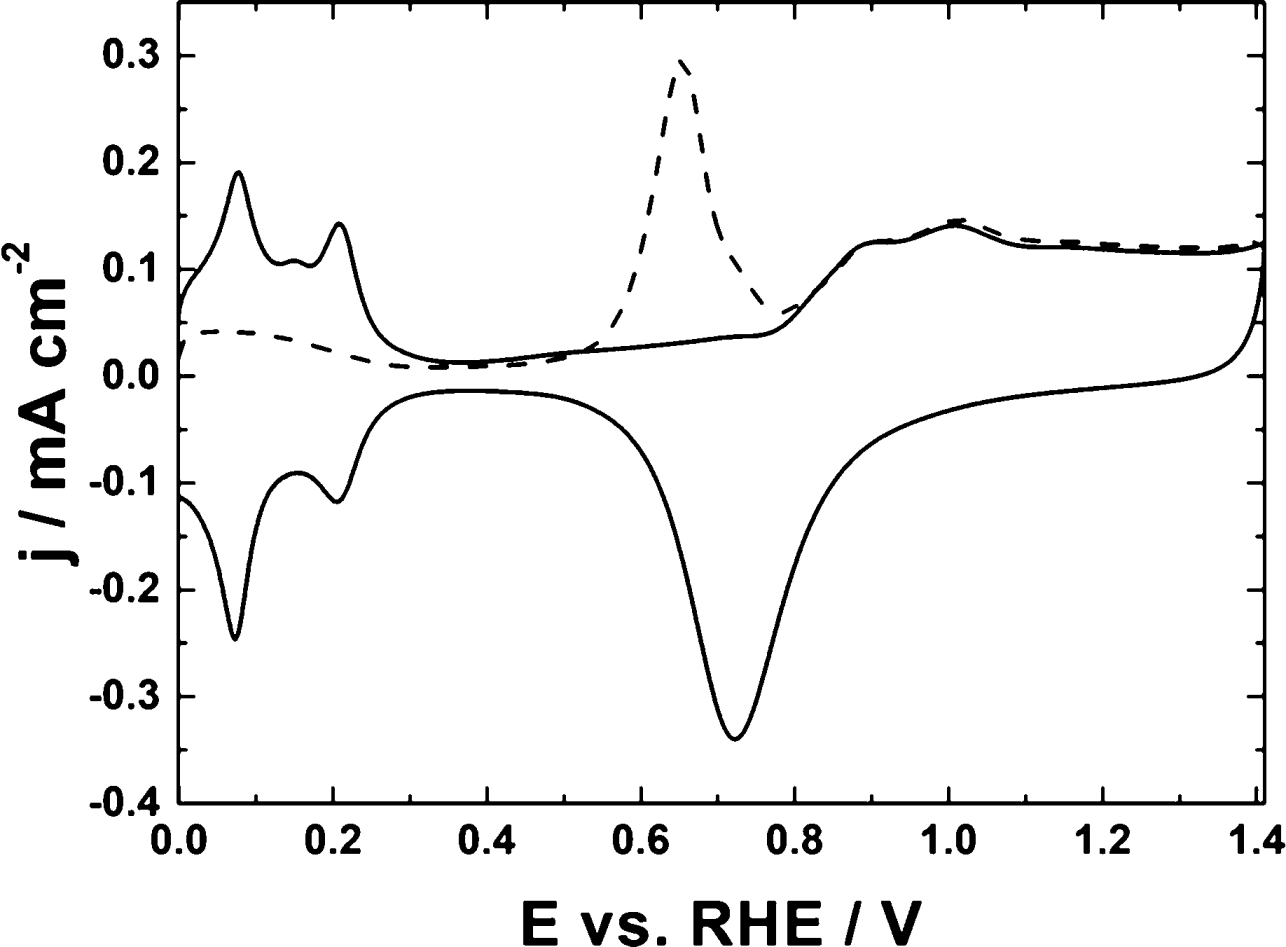
Stripping-type voltammetric oxidation (at 10 mV s^–1^) of the CO_2_R product (dashed line) which was adsorbed, or preconcentrated, on Pt nanoparticles deposited on the glassy carbon electrode, held at 0.34 V, and positioned adjacent to the Cu/WO_3_-modified working electrode at which the product had been generated upon application of the constant potential of −0.45 V for 2 h. Electrolyte, 0.5 mol dm^–3^ H_2_SO_4_. The experiment was executed in the bipotentiostatic mode.

When CO_2_R was carried out in deoxygenated acid solution at Cu/WO_3_, an independent stripping-type voltammetric diagnostic experiment was pursued in a manner analogous to that described previously.^90^ An additional working electrode with Pt catalytic nanoparticles (deposited on glassy carbon, as described earlier)^91^ was placed in the vicinity of the working electrode. After long-term (2 h) chronoamperometric-type (Figure 7C) reduction of CO_2_ at −0.45 V (performed in a two-chamber electrolytic cell subjected to continuous CO_2_ saturation), the reaction products were preconcentrated on surfaces of Pt nanoparticles (deposited onto the adjacent working electrode) upon application of 0.34 V for 1000 s. At this potential, which was more positive relative to those characteristics of the Pt surface hydrogen peaks, any CO_2_ existing in the reaction medium, even if exhibited some adsorptive interactions with Pt, did not undergo reduction. Thus, only CO_2_R products generated at the cathode were preconcentrated on Pt surfaces (at 0.34 V) of the additional electrode (placed nearby the working electrode). Later, this electrode was removed and transferred to deoxygenated 0.5 mol dm^–3^ H_2_SO_4_ to execute the stripping-type voltammetric oxidation step experiment (Figure 8). The solid black line reflected a typical response of Pt nanoparticles. In the potential range from 0 to 0.25 V, characteristic hydrogen adsorption peaks were developed, and at potentials higher than 0.85 V, the oxidation of platinum to platinum oxides (followed by their reduction peak below 0.8 V in the reverse scan) became operative. In between hydrogen peaks and the formation of Pt oxides, platinum exists mostly as Pt^0^ (so-called the double-layer region).^92^ The stripping-type voltammetric response of the CO_2_R product, which had been generated at the hybrid Cu/WO_3_-modified working electrode but adsorbed, or preconcentrated, on Pt nanoparticles (deposited on the adjacent glassy carbon electrode), yielded a single peak at about 0.65 V in the H_2_SO_4_ electrolyte (Figure 8A, dashed line). Such a voltammetric pattern could be attributed to the oxidation of the methanol adsorbate, where oxidation proceeded via the CO intermediate.^92^ Although the oxidation of CH_3_OH and CO species could not be distinguished, it should be remembered that no carbon monoxide as the gaseous bulk product was detected during chromatographic experiments. The result is not unequivocal because signals originating from the oxidation (on Pt) of other small organic molecules (ethanol, acetaldehyde) may overlap, while the presence of methanol may give rise to the stripping signal. On the other hand, our diagnostic experiments (Supporting Information) clearly show that a single fairly narrow adsorbate peak in the stripping voltammogram of Figure 8 would be accompanied by a prepeak (Figure S8) if—in addition to methanol (at 0.01 mol dm^–3^)—ethanol (even at the level as low as 1 mmol dm^–3^) were present in the electrolyte (before the medium transfer to 0.5 mol dm^–3^ H_2_SO_4_, in which the stripping voltammogram was recorded). The stripping response originating from acetaldehyde is also in the form of two overlapping peaks, and the analogous response of the formic acid adsorbate appears at much more positive potentials (Figure S8). Finally, when the stripping voltammetric experiment of Figure 8 has been repeated using Pd (rather than Pt) nanoparticles (deposited on glassy carbon), no stripping oxidation peak has been observed. The result is not surprising because Pd does not exhibit electrocatalytic properties toward the oxidation of methanol in the acid medium (Figure S9A). If formic acid was present in the medium, one would observe the oxidation peak in the potential range of 0.2–0.3 V (Figure S9B). Development of the latter peak is not significantly suppressed by the excess of methanol. In other words, no formic acid is expected as the CO_2_R reaction product here. Although the present data do not provide unequivocal evidence that methanol is the only C_*x*_H_*y*_O_*z*_-type reaction product, its dominating existence is very likely. The latter statement is in agreement with the results of chromatographic investigations.

### 3.4 XPS Analysis

To get further insight into the structure and chemical nature of copper sites and tungsten oxide (nanowires), XPS diagnostic experiments have been performed. Technical details are provided in the Experimental Section and elsewhere.^93^ The appearance of peaks in the range of 942–945 eV implies the presence of Cu^II^ sites on surfaces of the catalyst.^94^ This result (for simplicity not shown here) is not surprising because the samples have been exposed to air. The peak of Cu 2p_3/2_ having a BE of 932.6 eV corresponds to copper (I) (e.g., Cu_2_O) species. Copper (I) oxo species are expected to exist at the interfaces with tungsten oxide even following reduction under conditions of electrochemical experiments. Obviously, similarity of binding energies characteristic of Cu and Cu_2_O complicates somewhat characterization.^94^ The appearance of O 1s peaks at 530.4 eV is consistent with the existence of the oxygen ionic state in metal oxides, WO_3_, CuO, or Cu_2_O.^95,96^

**Figure 9:**
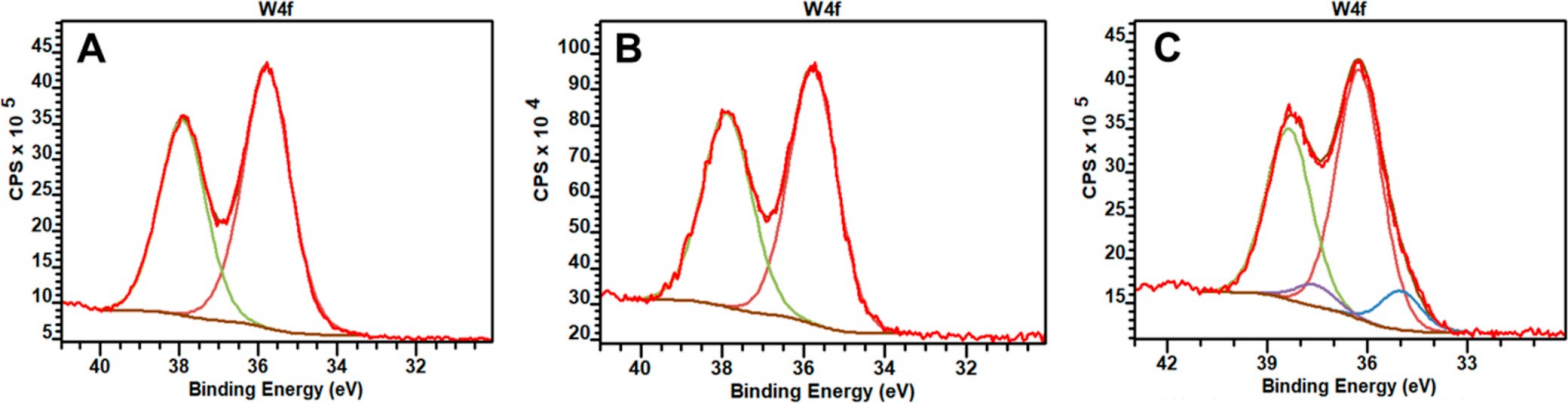
XPS spectra of W 4f of Cu/WO_3_ taken after electroreduction for 200 s at −0.45 V in 0.5 mol dm^–3^ H_2_SO_4_ in the (A) absence and (B) presence of CO_2_. (C) For comparison, the W 4f spectrum of prereduced WO_3_ admixed with Pt nanoparticles.

To comment on nonstoichiometry and the existence of oxygen vacancies or defects in tungsten oxide, the XPS spectra of the W 4f have been considered. Figure 9 illustrates main and shoulder peaks in W 4f XPS spectra of Cu/WO_3_ taken after electroreduction (for 200 s at −0.45 V) in 0.5 mol dm^–3^ H_2_SO_4_ in (A) the absence and (B) the presence of CO_2_. For comparison, (C) the W 4f spectrum of prereduced WO_3_ admixed with Pt nanoparticles is provided. In the latter case, the formation of hydrogen tungsten (VI, V) oxide bronzes was well established.^97^ The XPS data are largely similar for both Cu/WO_3_ samples reduced in the absence and presence of CO_2_. For the deconvolution curve, appearance of the peaks at 35.61 eV shall be attributed to W 4f_7/2_, which corresponds to the W^VI^ oxidation state existing in Cu/WO_3_ referring to W 4f_3/2_.^98,99^ However, the energetic position of W 4f_5/2_ (red component) implies existence of both W^IV^O_2_ and W^VI^O_3_ (i.e., W^VI,IV^O_3–*y*_, with electrons localized at W^VI^ and W^IV^ sites), rather than only H_*x*_W^VI,V^O_3_ (with electrons delocalized over W^VI^ and W^V^), as expected for the prereduced Pt/WO_3_ (blue component with ca. 0.4 eV shift). The result is consistent with the formation of the oxygen-deficient W^VI,IV^O_3–*y*_ species during electroreductions. The reduced W^IV^ sites are likely to be stabilized by coexisting Cu species.

### 3.5 Importance of Tungsten Oxide as a Copper Cocatalyst

**Figure 10:**
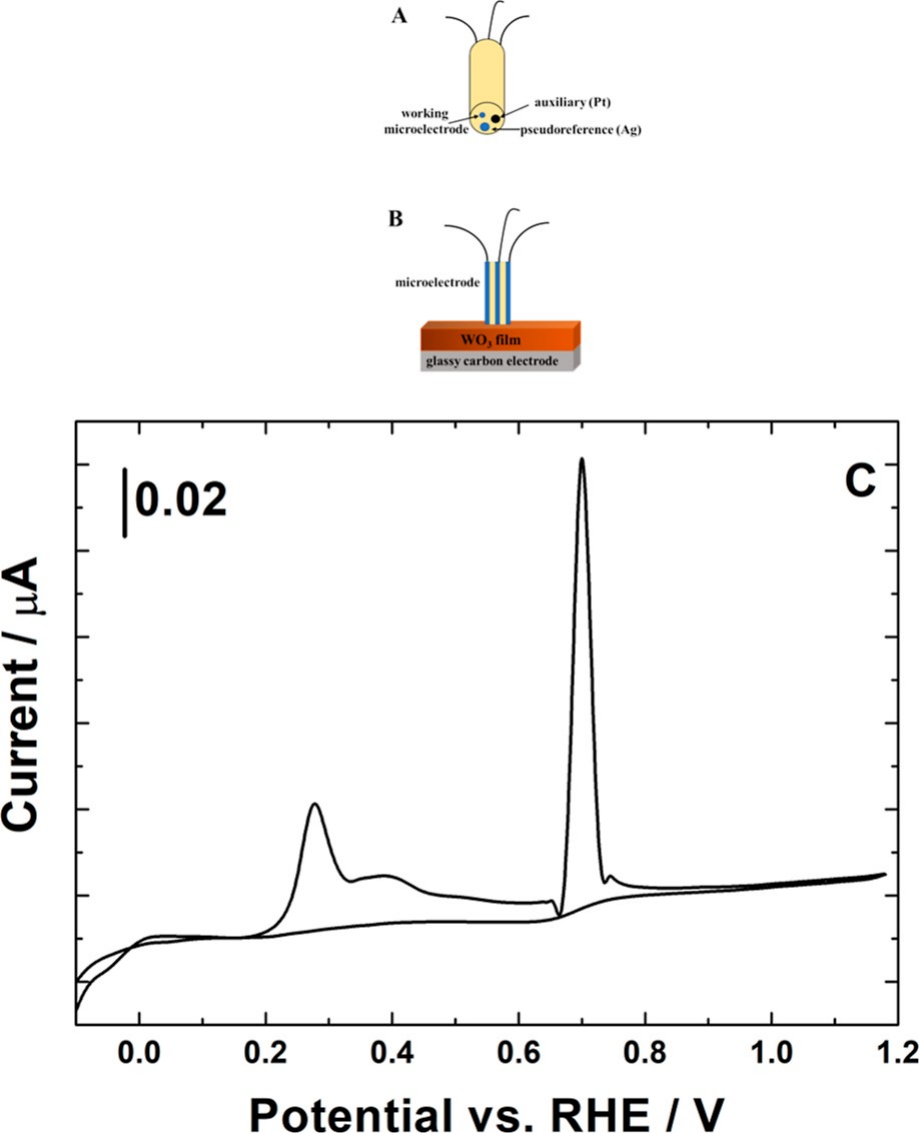
(A) Schematic diagram of the ultramicroelectrode-based probe (assembly) with a planar three-electrode configuration. (B) Scheme illustrating the necessity of close contact between the WO_3_ film and the electrodes in planar configuration. (C) Stripping-type voltammetric experiment (performed in deaerated 0.5 mol dm^–3^ H_2_SO_4_ at 10 mV s^–1^ scan rate) using the three-electrode assembly touching the film of WO_3_ (prereduced to H_x_WO_3_ at −0.3 V for 300 s in the presence of CO_2_). Diameter of the Pt ultramicrodisk electrode, 20 μm.

Tungsten oxide is known to exhibit considerable gas sensitivity toward carbon monoxide, which undergoes moderate (physical) adsorption on WO-terminated and fairly strong chemisorption on O-terminated surfaces of hexagonal tungsten oxide.^100,101^ The strong CO_2_-adsorption capacity of tungsten oxides has also been postulated. To comment about reactivity and possible interactions between the partially reduced WO_3_ nanorods (mostly to hydrogen tungsten bronzes, H_*x*_WO_3_) and carbon dioxide, we performed the following electrochemical diagnostic experiments. First, a film of Cu-free WO_3_ nanorods (deposited on glassy carbon) was reduced at −0.45 V for 120 s in CO_2_-saturated 0.5 mol dm^–3^ H_2_SO_4_. Following rinsing with distilled/deionized water, the electrode was transferred to a glass cell (filled with deoxygenated 0.5 mold dm^–3^ H_2_SO_4_) and positioned opposite the Pt-ultramicroelectrode-disk-containing planar three-electrode probe (Figure 10A) historically developed for solid-state voltammetry.^102−105^ Care was exercised to assure contact between the film and the electrodes (Figure 10B) as well as to keep the oxygen-free nitrogen-saturated atmosphere in the cell. Figure 10C illustrates the stripping-type voltammetric response recorded at the Pt ultramicrodisk electrode touching the layer of WO_3_ nanorods (deposited onto the surface of the opposing glassy carbon electrode). The appearance of the sharp stripping peak at about 0.7 V should be correlated with the oxidation of CO (that apparently was generated as an adsorbate on H_*x*_WO_3_ nanostructures) and detected at the contacting Pt ultramicroelectrode.^92,106^ Furthermore, it is apparent from the voltammetric stripping data that H_2_ oxidation is accompanied by CO oxidation in the potential region below 0.4 V, as postulated elsewhere.^106^ The latter phenomenon seems to reflect the Pt-induced activity of the W^VI/V^ redox couple toward CO oxidation in the acid medium. Because the stripping voltammogram was initiated at −0.1 V, where hydrogen evolution could be operative, it is not surprising that the dominating H_2_-oxidation peak at about 0.28 V was observed. As a consequence of the overlapping, the W^V^ (tungsten bronze)-driven oxidation of CO was somewhat shifted to more positive potentials (but still below 0.4 V).^106^ The appearance of a broad peak at about 2100 cm^–1^ during FTIR examination (by reflectance) in the spectrum of tungsten oxide prereduced in the presence of CO_2_ (Figure S10), relative to its absence in the spectrum of the system considered in the absence of CO_2_, seems to support the hypothesis about the feasibility of sorption of CO_2_ or its CO-type reduction products.^106^ Also, the XPS results are consistent with the view about the strong affinity of WO_3_ and Cu/WO_3_ toward CO_2_, CO, or the CO_2_R intermediates.

The present data clearly imply the ability of WO_3_ to absorb hydrogen as well as the strong affinity of WO_3_ toward adsorption of CO_2_ or CO-type reaction intermediates. These are surface phenomena because no CO gas was detected using GC-MS as the reaction product. While the ability of WO_3_ to absorb hydrogen would explain the better tolerance of the Cu/WO_3_ catalyst with respect to the competitive hydrogen evolution during CO_2_R, the capacity of WO_3_ to exhibit adsorptive interactions toward CO should facilitate removal of the poisoning CO-type adsorbates from Cu catalytic sites.

### 3.6 Charge Propagation and Trapping in Bi-Component Cu/WO_3_

**Figure 11:**
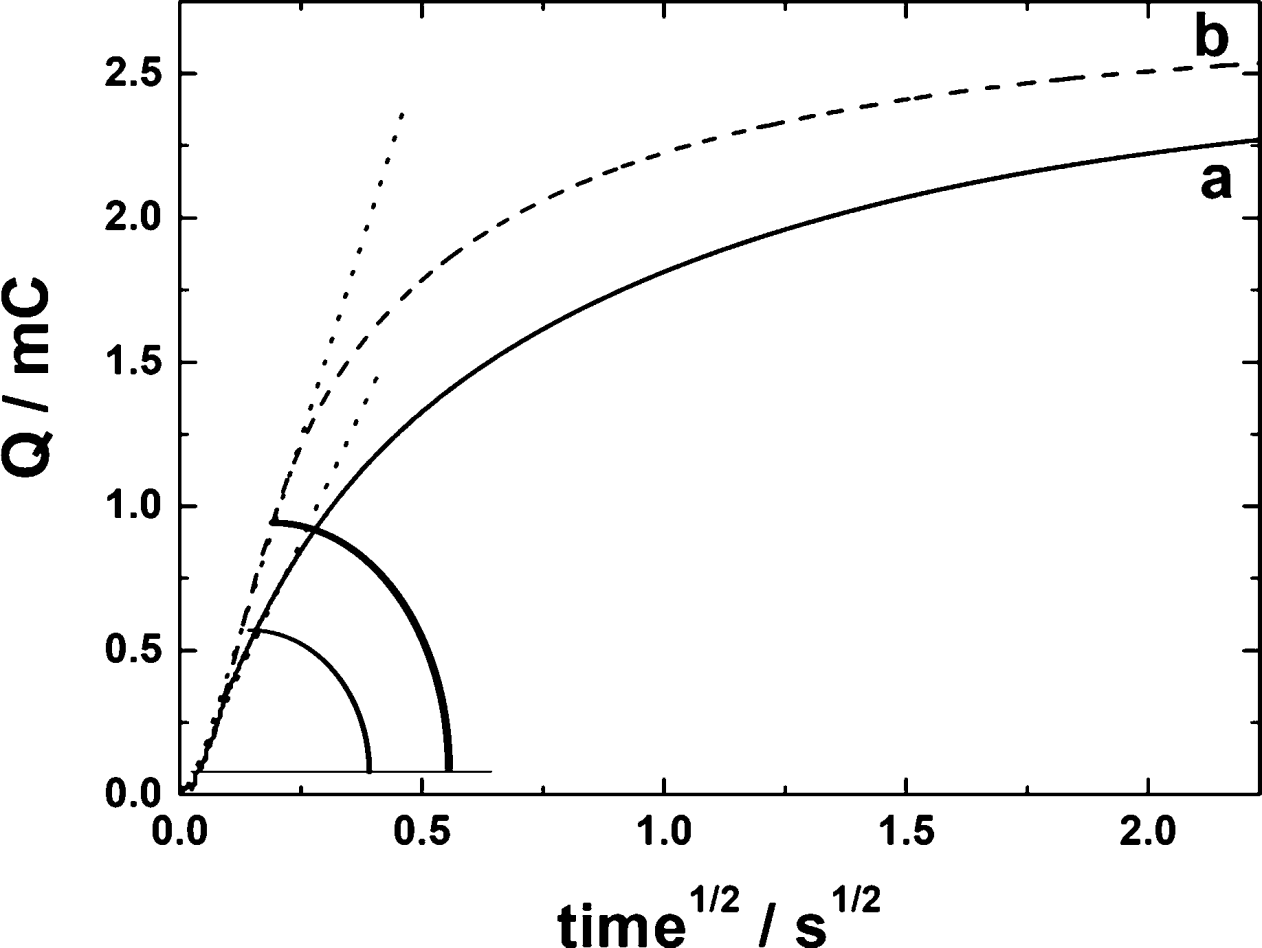
Chronocoulometric responses recorded at the glassy carbon electrode modified with (a) pristine WO_3_ nanowires and (b) Cu/WO_3_ film. Potential steps (5 s) were from 0.6 to −0.3 V. Electrolyte, 0.5 mol dm^–3^ H_2_SO_4_.

Despite specific activity and selectivity, useful electrocatalytic systems are characterized by fast charge propagation that enables facile electrical communication with an inert reactant (here CO_2_). In the case of tungsten oxide films, charge transport can be described in terms of the effectively diffusional electron transfers (redox transitions leading to the reversible formation of hydrogen bronzes) compensated readily by the proton displacements.^74^ For such redox-conducting films,^107^ chronocoulometry appears a good diagnostic tool^74,103,104^ and permits to comment about dynamics of charge transport under effectively diffusional conditions. Figure 11 illustrates the results of the potential step measurements (from 0.6 to −0.3 V) covering tungsten oxide redox transitions for both sets of peaks (Figures 3A and 5B), in which charge (Q) has been accumulated during the reduction of (a) Cu-free and (b) Cu-containing WO_3_ films and plotted versus square root of time. Two characteristic domains can be distinguished in both plots (a) and (b) of Figure 11. For shorter times (i.e., at time^1/2^ values below 0.5 s^1/2^), charge propagation can be described in terms of a semi-infinite diffusion model where Q versus time^1/2^ dependencies develop linear portions with effectively zero intercepts. For longer times (i.e., at time^1/2^ values above 1.5 s^1/2^), thin-layer-type saturation of charge becomes operative, and the Q values tend to reach plateau responses due to practically complete reductive electrolysis of films.

The linear (effectively diffusional-type) portions of chronocoulometric plots (Figure 11; plots a and b) can be analyzed according to integrated Cottrell equation^108^
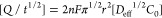
1where *r*, *D*_eff_, and *C*_0_ stand for the electrode radius, the effective (apparent) diffusion coefficient for charge propagation, and the concentration of redox centers, respectively; the other parameters have their usual significance.^108^ Because of uncertainty in the estimation of W^VI,V^ mixed-valent redox centers (*C*_0_), determination of the charge transport parameter, *D*_eff_^1/2^*C*_0_ (rather than simply *D*_eff_), has been pursued here. It should be emphasized that the *D*_eff_^1/2^*C*_0_ parameter appears in equations describing current responses in such common controlled-potential electrochemical methods as voltammetry or chronoamperometry.^108^ The linear portion of the plot for the Cu/WO_3_ film (curve b) is characterized by a higher slope (4.85 mC s^–1/2^) relative to that characteristic of the Cu-free WO_3_ film (3.51 mC s^–1/2^) (curve a). Assuming the one-electron nature of transfers between W^VI^ and W^V^ sites, the following *D*_app_^1/2^*C*_0_ values have been determined: (a) 4.6 × 10^–7^ and (b) 6.3 × 10^–7^ mol cm^–2^ s^–1/2^. Knowing that common redox-polymer-type electroactive systems^77,102,103^ are characterized by *D*_app_’s from 1 × 10^–8^ up to 5 × 10^–7^ cm^2^ s^–1^, *C*_0_’s from 0.2 up to 2.0 × 10^–3^ mmol cm^–3^ and, consequently, by *D*_app_^1/2^*C*_0_ parameters ranging from 2 × 10^–8^ to 1 × 10^–6^ mol cm^–2^ s^–1/2^, the obtained *D*_app_^1/2^*C*_0_ values here for both Cu-free WO_3_ and Cu/WO_3_ systems are consistent with the fairly fast charge transport mechanism. The fact that the *D*_app_^1/2^*C*_0_ charge transport parameter has somewhat increased upon incorporation of Cu into hexagonal WO_3_ may reflect the ability of the dispersed metallic copper sites to facilitate electron transfers and charge distribution within the catalytic layers.

Finally, some attention has been paid to our unique observation that by subjecting the bilayer film of Cu_2_O over-coated with WO_3_ to voltammetric potential cycling in 0.5 mol dm^–3^ H_2_SO_4_ (Figure 5A), copper has been incorporated within tungsten oxide (Figure 6), and the resulting Cu/WO_3_ film contained basically nonelectroactive copper sites in the fairly broad range of potentials (Figure 5B). To explain this phenomenon, we refer to the historic concept of charge trapping (storage) in bi-layer-type coatings on electrodes.^109^ Here, the Cu/WO_3_ system can be considered as the bicomponent assembly in which copper ions once incorporated within hexagonal WO_3_, subsequently reduced, mostly, to Cu^0^ (at potentials where WO_3_ undergoes reduction to hydrogen bronzes, H_*x*_WO_3_),^74^ cannot be reoxidized because the potential for the reoxidation of H_*x*_WO_3_ to WO_3_ is not positive enough to drive, or mediate, the oxidation of copper ions (compare potentials of the respective redox transitions in Figure 3A and in the inset of Figure 2). It is reasonable to expect that the trapped copper sites are surrounded by WO_3_, and they do not have direct access to the electrode surface. Such behavior can be achieved only when copper sites are strongly attached to or coordinated by WO_3_ or incorporated to WO_3_. A tempting and possible explanation takes into account integration of copper species within the structural channels of hydrous hexagonal WO_3_.^83^ In addition to Cu^0^, some of copper sites may exist as sparingly soluble Cu(I) oxo species stabilized by the tungstate network. Nevertheless, the tungsten oxide host does not seem to be thermodynamically capable of mediating the reoxidation of Cu sites, thus supporting their trapping within WO_3_. Obviously, the charge injected during reduction of copper ions immobilized within the WO_3_ structure should also be trapped (rather irreversibly) during cathodic polarization. If it is the case, unique electrocatalytic reductive properties, in addition to improved conductivity, can be expected. Further research is needed along this line.

### 3.7 Efficiency, Durability, and Dynamics of CO_2_R

Our present results are consistent with the view that methanol is likely the main, or at least the dominating, C_*x*_H_*y*_O_*z*_-type oxo-hydro-carbon reaction product, the formation of which is accompanied by evolution of hydrogen. However, it should be remembered that the formation of H_2_ is the two-electron reaction, and conversion of carbon dioxide to methanol is the six-electron process. The estimation of Faradaic efficiency for the methanol formation has been based not only on the above assumptions but also on correlation of charge passed during electrolysis with the amount of moles of the generated product (methanol). Following the long-term (7200 s) CO_2_R controlled potential (−0.45 V) electrolysis (in two-chamber cell) experiment in the deoxygenated CO_2_-saturated 0.5 mol dm^–3^ H_2_SO_4_ solution using the relatively large-geometric-surface-area (2 cm^2^) Cu/WO_3_-modified glassy carbon electrode, methanol has been determined as described in the Supporting Information (Figure S11). Judging from the amount (mol) of CH_3_OH in the reaction medium, 34 C, out of 50 C, that is, the total charge passed during electrolysis (Figure S12), has been assumed to be consumed to produce methanol (six-electron process). The remaining portion of charge (16 C) is attributed to hydrogen evolution (two-electron process). Thus, it can be rationalized that the Faradaic efficiency, which in a sense is the selectivity (molar) efficiency, toward production of methanol is on the level 42%. Furthermore, the yield rate has been estimated under such conditions (i.e., during the CO_2_R electrolysis at for 7200 s at the Cu/WO_3_-modified electrode of geometric area 2 cm^2^) and found to be equal to 15 μmol cm^–2^ h^–1^ (41 μmol m^–2^ s^–1^). Of course, due to some uncertainty in determination and identification of methanol as the main C_*x*_H_*y*_O_*z*_-type oxo-hydro-carbon reaction product, the above parameters are obviously estimates.

An attempt has also been made to differentiate and collate our results with data existing in the literature, particularly with the recent reports describing activity of various Cu- or Cu_2_O-based systems, including those utilizing metal–organic porous materials, heterometallic metal–organic frameworks, or bimetallic alloys for CO_2_R toward alcohols, primarily methanol.^116−122^ Despite the fact that our diagnostic experiments have been performed in a different medium (strong acid, rather than typically explored semi-neutral or alkaline electrolytes) as well as upon application of markedly less negative potentials (e.g., at −0.45 V vs RHE, i.e., at least 200–500 mV more positive, when recalculated to the same potential scale), the Faradaic efficiency (42%) and yield rate (41 μmol m^–2^ s^–1^) obtained here are comparable to those recently considered and tabularized (typically in the range from 15 to almost 50% and from 5 to almost 60 μmol m^–2^ s^–1^).^116^ Even if our parameters have been somewhat overestimated, simple comparison of voltammetric current densities reported here (Figure 7A,B) and reported elsewhere^116−122^ implies large similarity in performance (upon normalizing to the same scan rate) under conditions where CO_2_R processes are dominating versus the background hydrogen evolution process. Thus, our hybrid catalytic system seems to be well-behaved despite operating in the acid medium. Furthermore, stability of the dependence of current versus time recorded during CO_2_R under the conditions of long-term (2 h) electrolysis (−0.45 V) at the Cu/WO_3_-modified electrode (Figure S12A) is consistent with good durability of the electrocatalytic performance. Finally, the monotonic increase of negative charge (Figure S12B) during prolonged electrolysis also implies the overall system stability.

## 4 Conclusions

The present study shows that copper seems to be unique among metals not only in its ability to catalyze the electrochemical reduction of CO_2_ to distinct reductively more advanced products but also in potentiality to interact reactive metal oxides, such as WO_3_. The latter phenomenon has translated into selectivity against competitive hydrogen evolution, feasibility of pursuing CO_2_ electroreduction in an acid medium (without addition of any ions, such as potassium cations, as reported earlier^69,70^), and capability of obtaining methanol as the main CO_2_R reaction product. As an alternative to the postulated stabilization of CO_2_^–^ intermediates by partially dehydrated metal cations,^123^ the feasibility of local activating interactions between tungsten oxo species and the CO_2_R intermediates can be postulated. Further research is needed along this line. We have demonstrated that hierarchically deposited films (on glassy carbon) of copper (I) oxide decorated with tungsten (VI) oxide nanowires and, subsequently, subjected to electroreduction and voltammetric conditioning to generate the hybrid Cu/WO_3_ catalyst can be successfully utilized to drive reduction of carbon dioxide (saturated solution, concentration, ca. 0.033 mol dm^–3^)^110^ in a fairly strong acid medium of 0.5 mol dm^–3^ H_2_SO_4_. In this respect, our study parallels recent reports^69,70^ concerning CO_2_ electroreduction in acid media. Here, the formation of the partially reduced tungsten oxides (H_*x*_WO_3_ and WO_3–*y*_) is accompanied by consumption of protons and sorption of hydrogen, and it tends to inhibit hydrogen evolution by shifting the proton discharge toward more negative potentials, which seems to be of importance here. Our observations are consistent with the view that copper is irreversibly trapped within the network of WO_3_ nanowires. The dispersed metallic copper sites seem to facilitate electron transfers and charge distribution in the catalytic layers. Among important issues are the capacity of partially reduced tungsten oxides to sorb CO_2_- and CO-type reaction intermediates as well as the possibility of activating interactions between tungsten oxo and hydroxo surface groups and copper catalytic centers. Under such conditions, the oxidative desorption of CO from the Cu sites existing within the WO_3_ matrix becomes feasible. On mechanistic grounds, the existence of hydrogen-rich partially reduced tungsten oxides, H_*x*_WO_3_, which contain a large population of delocalized electrons and monoatomic H, or coexisting protons and electrons, H^+^ + e^–^, is likely to induce hydrogenation of CO-type adsorbates on Cu to form −CH_2_OH intermediates, followed by protonation.^41^ The hybrid Cu/WO_3_ system, while exhibiting better selectivity toward CO_2_ reduction, relative to hydrogen evolution (proton discharge), is also characterized by improved durability, relative to pristine Cu_2_O-derived copper, as evident from chronoamperometric CO_2_R. Inspired by our preliminary results,^111^ further research aiming at optimization of the material’s activity and selectivity could concentrate on application of mixed oxides, for example, WO_3_–ZrO_2_. Finally, the present study demonstrates the usefulness of certain diagnostic electroanalytical approaches, such as ultramicroelectrode-based sensing,^102−104,112^ chronocoulometric probing of the diffusional-type charge propagation dynamics,^77,102,108^ or voltammetric stripping and monitoring of small organic molecules.^85,90,113−115^

## Supporting Information Available

Experimental section for addition experiments, electrochemical measurements, XRD, SEM, CO_2_RR product analysis, stability and electrocatalytic durability, long-term electrolysis, voltammetric stripping, and monitoring of small organic molecules (PDF)
