# Supplementary material for: Toward Effective CO2 Reduction in an Acid Medium: Electrocatalysis at Cu2O-Derived Polycrystalline Cu Sites Immobilized within the Network of WO3 Nanowires
Source: ACS Meas Sci Au. 2022 Jun 28;2(6):553–67. doi: 10.1021/acsmeasuresciau.2c00010 (PMC9885951; doi:10.1021/acsmeasuresciau.2c00010)
Supplement: Supplementary file 1 — tg2c00010_si_001.pdf [file tg2c00010_si_001.pdf]

SUPPORTING INFORMATION

Toward Effective CO_2-Reduction in Acid Medium: Electrocatalysis at Cu_2O-Derived Polycrystalline Cu Sites Immobilized within Network of WO_3 Nanowires

Iwona A. Rutkowska*[†], Anna Chmielnicka[†], Maciej Krzywiecki[‡], Pawel J. Kulesza*[†]

[†]*Faculty of Chemistry, University of Warsaw, Pasteura 1, PL-02-093 Warsaw, Poland*

[‡]*Silesian University of Technology, Institute of Physics–CSE, Konarskiego 22B, 44-100 Gliwice, Poland*

Corresponding Authors: P.J. Kulesza (pkulesza@chem.uw.edu.pl)

I.A. Rutkowska (ilinek@chem.uw.edu.pl)

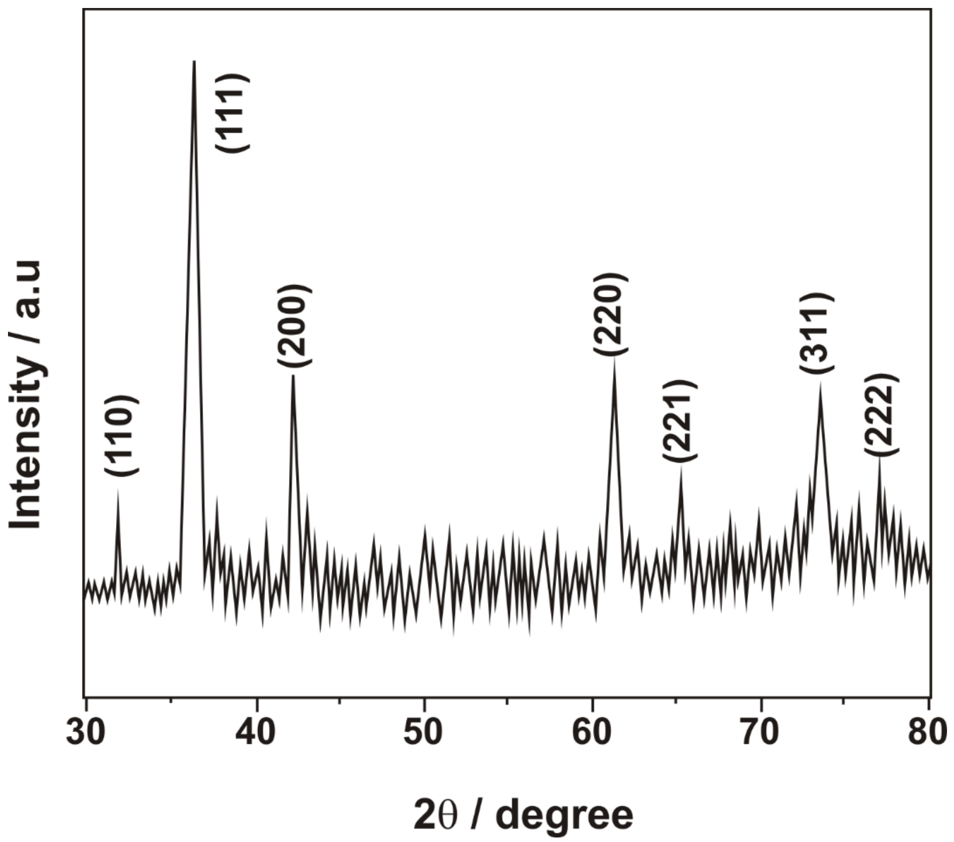

Figure S1. XRD of Cu_2O nanoparticles.

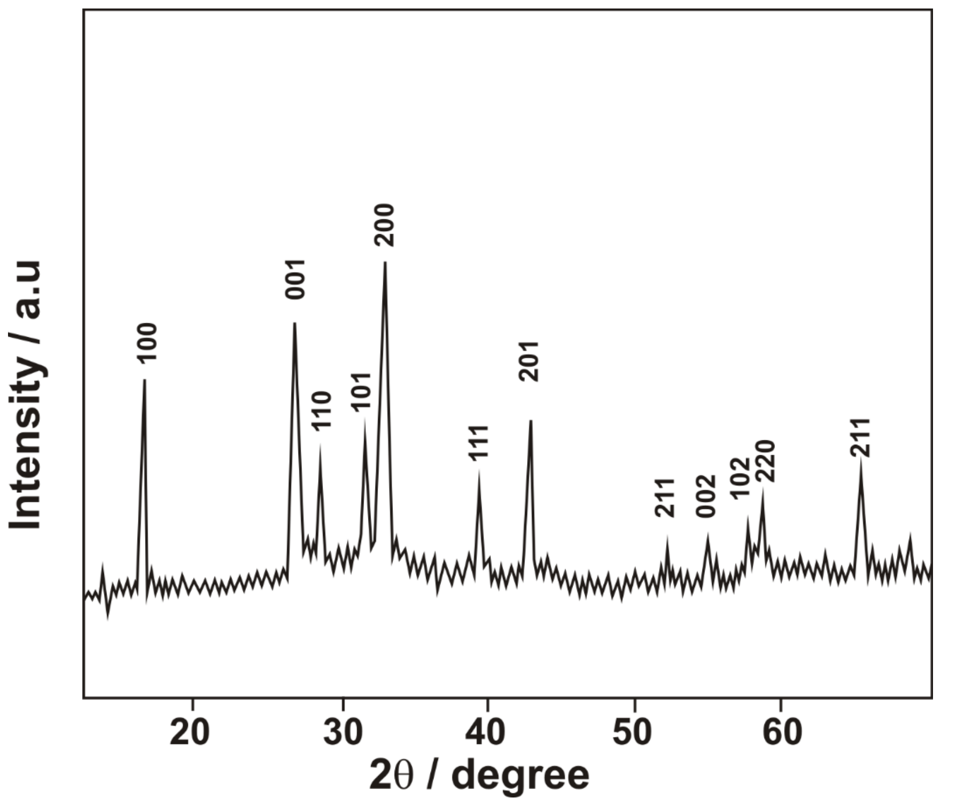

Figure S2. XRD of WO_3 nanowires.

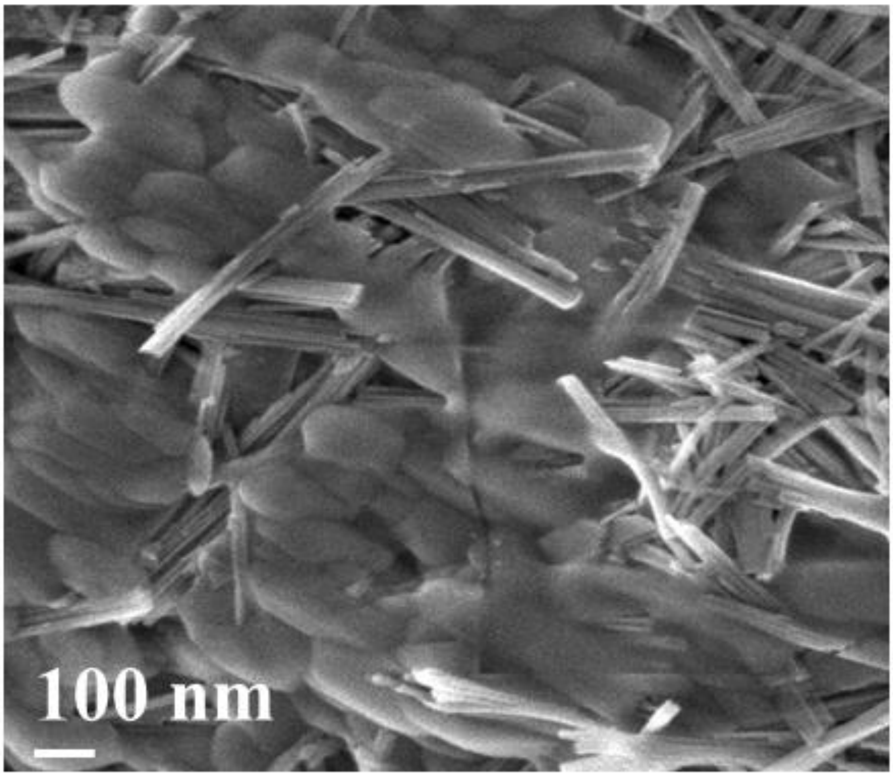

Figure S3. Scanning electron micrographs of hybrid system Cu/Cu_2O over-coated with WO_3.

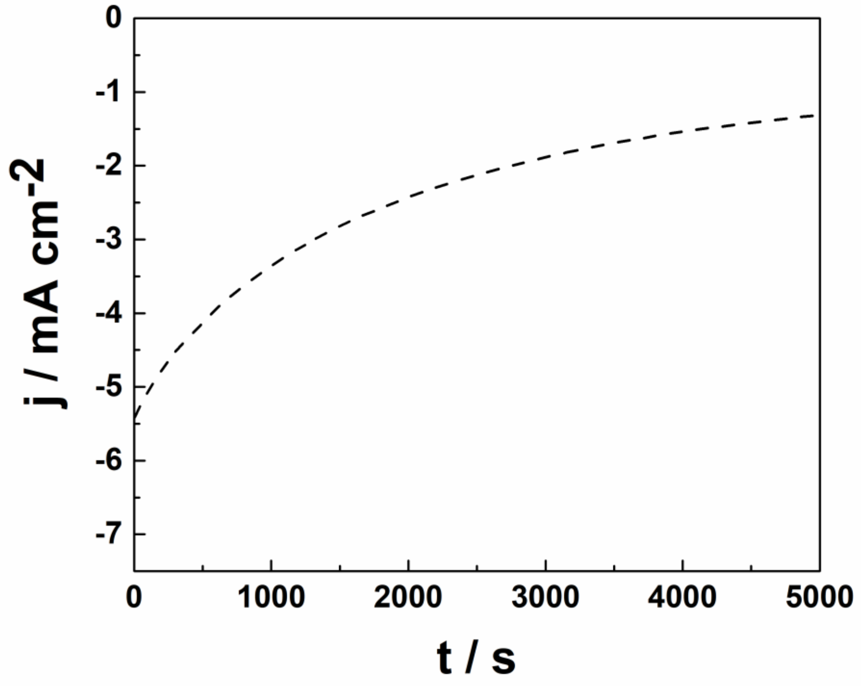

Figure S4. Chronoamperometric responses recorded upon application -0.45 V during reduction of CO_2 at pristine Cu_2O-derived Cu (dotted line). Electrolyte, 0.5 mol dm^{-3} H_2SO_4. Due to large contribution of hydrogen evolution, no attempt has been made to subtract the background currents (recorded in the absence of CO_2).

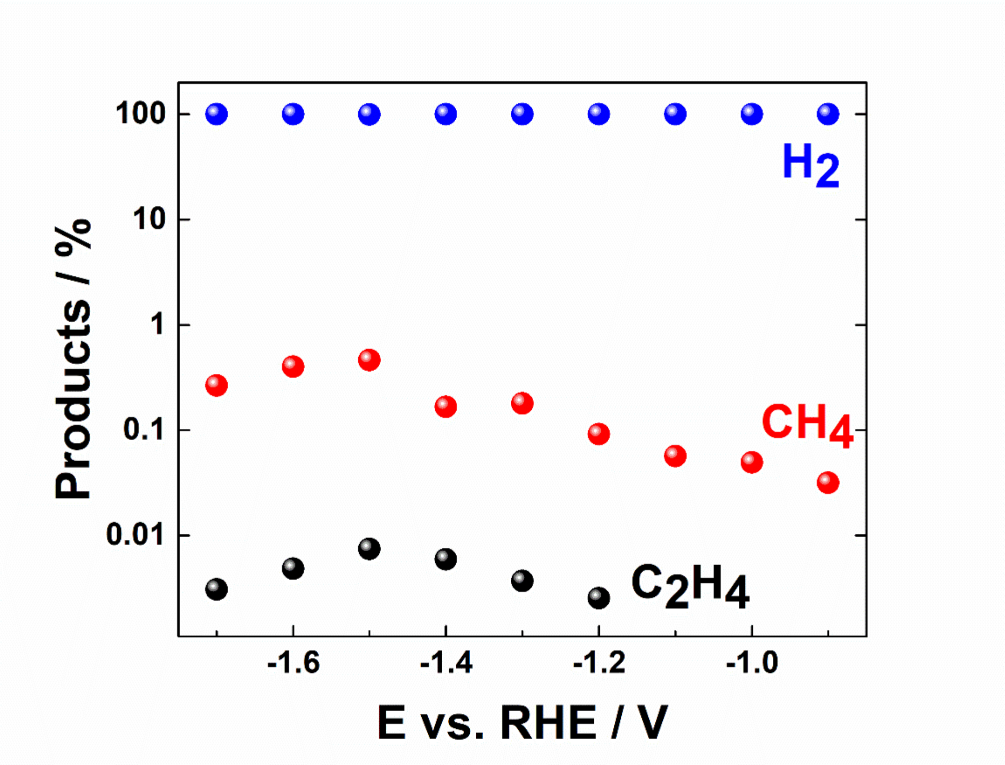

Figure S5. Distribution of gaseous products upon application of potentials more negative than -0.9 V.

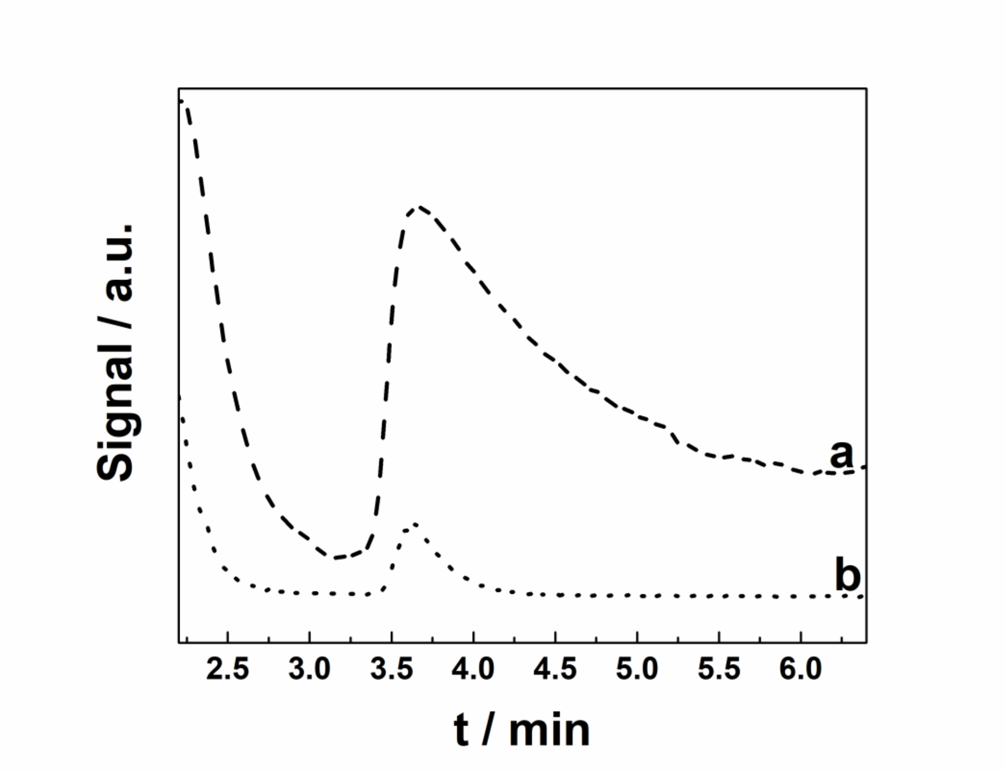

Figure S6. GC-AED chromatograms (obtained using HP-Innowax column) and recorded for (**a**) standard methanol sample, and (**b**) methanol trapped as volatile product following long-term electrolysis at -0.45 V.

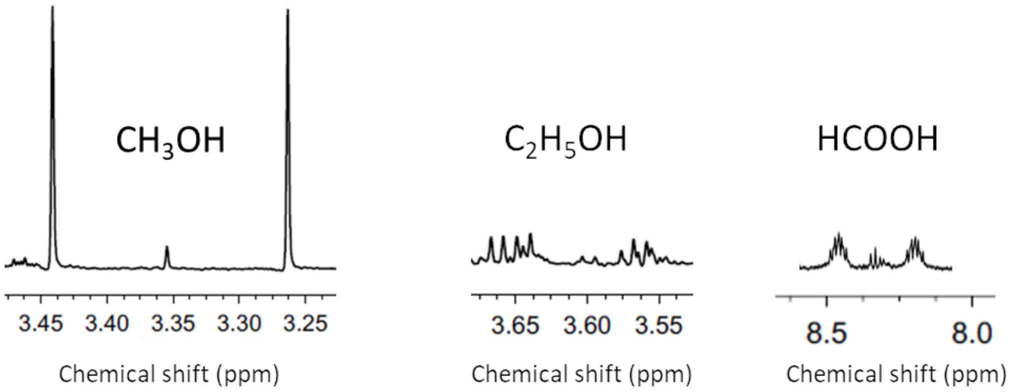

Figure S7. ^{1}H NMR spectra (taken at different chemical shifts/ppm) implying the presence of methanol in the sample after long-term electrolysis.

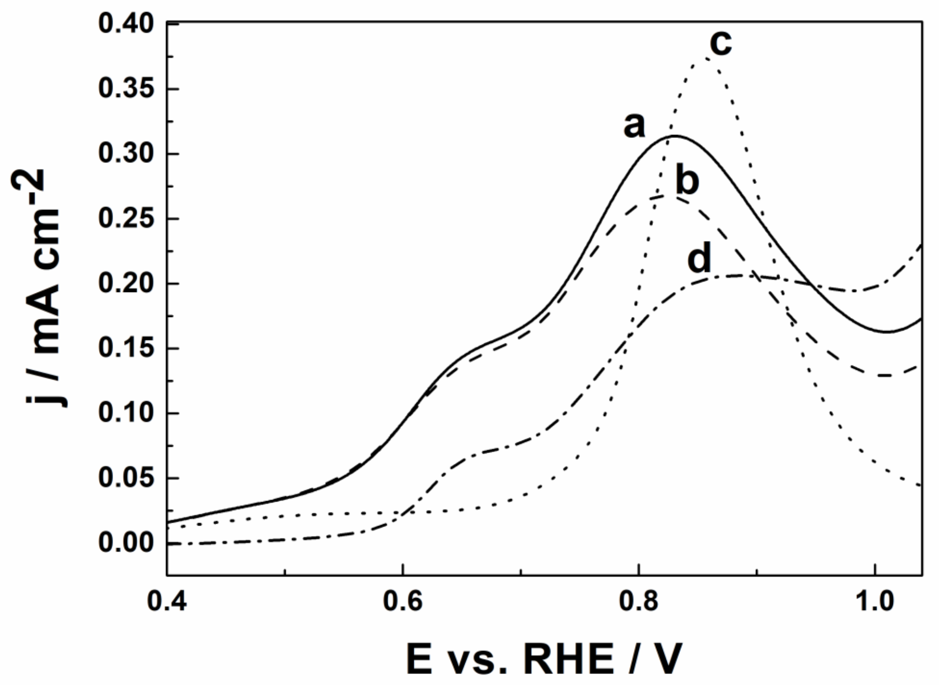

Figure S8. Stripping-type voltammetric responses taken during oxidation of adsorbates formed upon application of 0.34 V for 5 min to the Pt electrode in (a) 0.01 mol dm^{-3} methanol + 0.001 mol dm^{-3} ethanol, (b) 0.001 mol dm^{-3} ethanol, (c) 0.001 mol dm^{-3} formic acid, and (d) 0.001 mol dm^{-3} acetaldehyde . Electrolyte: nitrogen-saturated 0.5 mol dm^{-3} H_2SO_4. Scan rate, 10 mV s^{-1}.

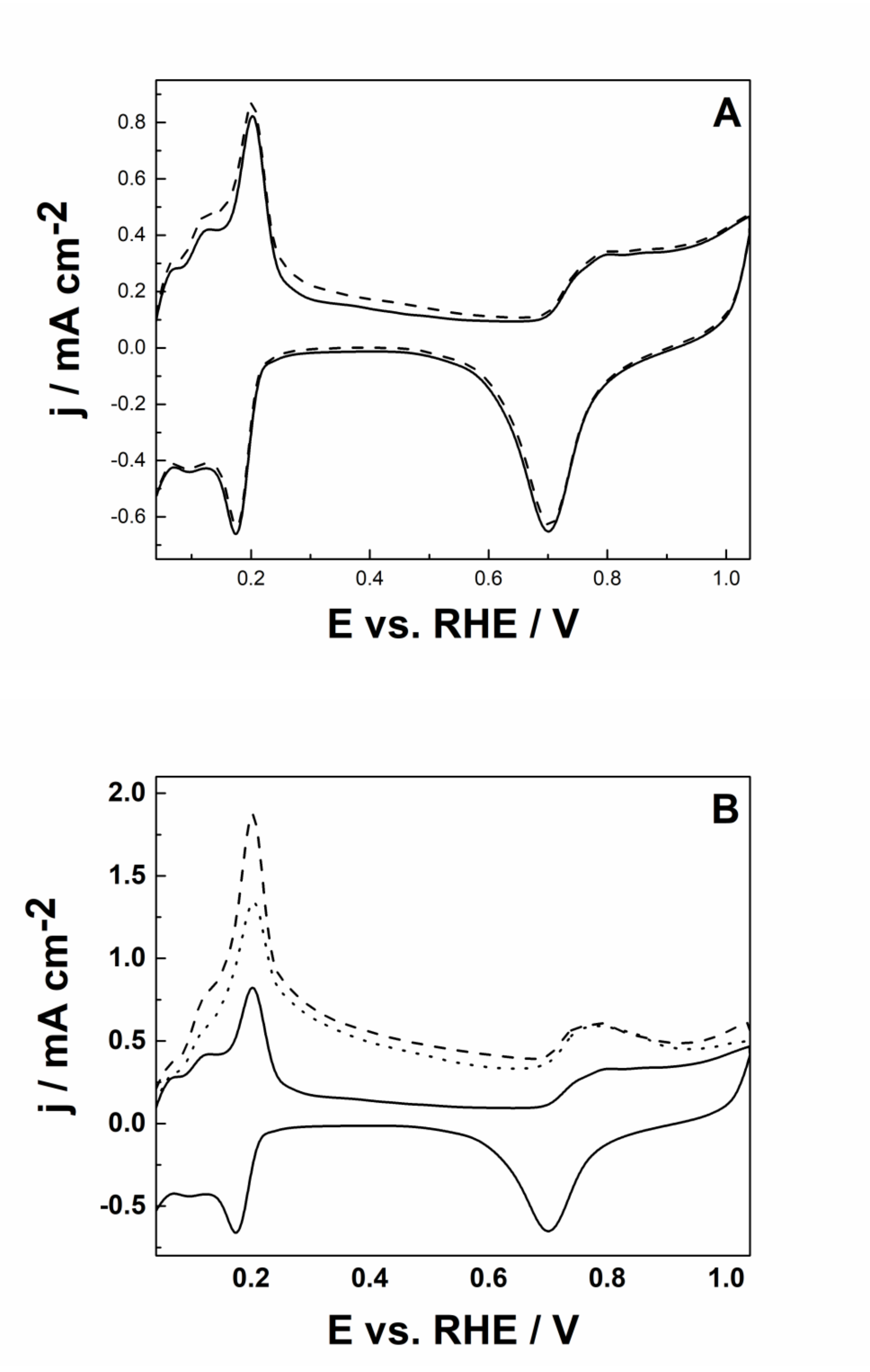

Figure S9. (A) Cyclic voltammetric responses recorded for Pd nanoparticles (deposited on glassy carbon at 100 μg cm^{-2} loading) in the presence (dashed line) and the absence (solid line) of methanol (0.5 mol dm^{-3} CH_3OH). Electrolyte: nitrogen-saturated 0.5 mol dm^{-3} H_2SO_4. Scan rate, 10 mV s^{-1}. (**B**) Voltammetric oxidation of formic acid (0.01 mol dm^{-3} HCOOH) in the absence (dashed line) and in the presence (dotted line) of methanol (0.5 mol dm^{-3} CH_3OH). Solid line stands for the cyclic voltammetric response of Pd nanoparticles (deposited on glassy carbon at 100 μg cm^{-2} loading) in nitrogen-saturated 0.5 mol dm^{-3} H_2SO_4. Scan rate, 10 mV s^{-1}.

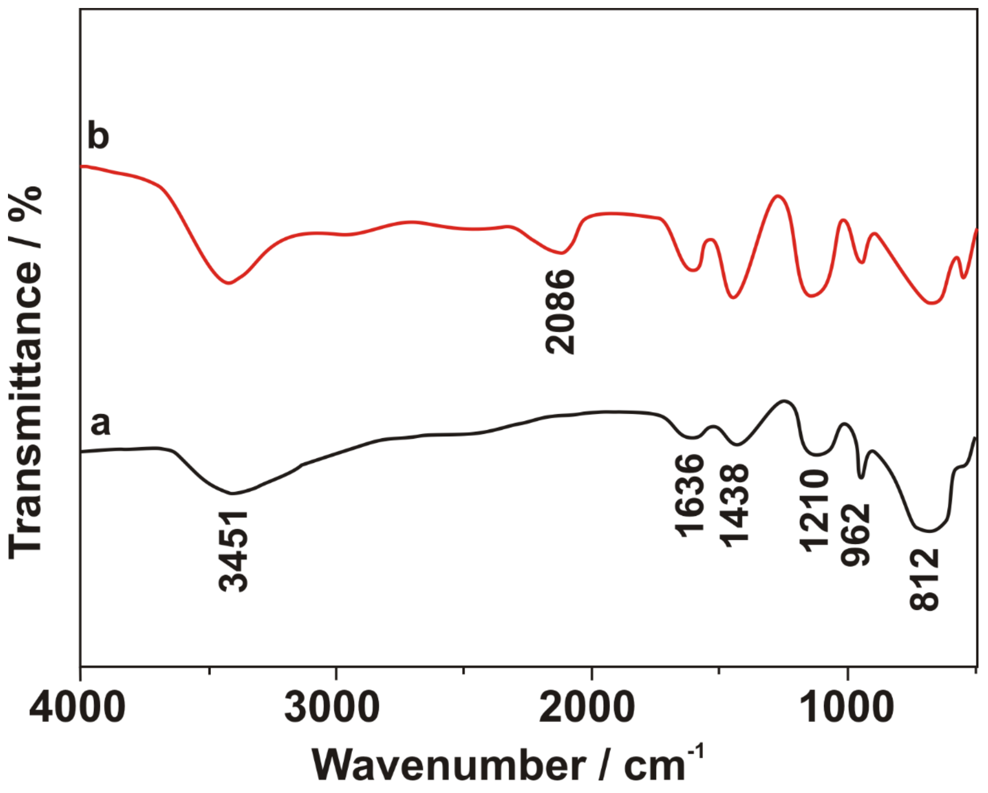

Figure S10. FTIR spectrum (by reflectance) of (**a**) pristine WO_3 nanorods, and (**b**) WO_3 nanorods subjected to electroreduction in the presence of CO_2 (as for Figure 10).

Electroanalytical Determination of Methanol

The analytical concept has been based on our previous obervations postulating proportionality of the PtRu-induced methanol-oxidation currents (recorded in 0.5 mol dm^{-3} H_2SO_4 electrolyte) on CH_3OH concentration as well as on the feasibility of determinations of methanol in the mmol dm^{-3} (or even lower) range of concentrations. In addition to a need of detection and quantification of low concentrations of toxic methanol historically, electrooxidation of CH_3OH attracted broad interest with respect to possible applications in direct methanol fuel cells utilizing proton-exchange membranes. In this context, a concept of the electrooxidation of CH_3OH to CO_2 has been widely explored despite the complexity of the mechanism for oxidation of methanol.

Figure S11A illustrates results of a series of blank CH_3OH-oxidation voltammetric experiments (Curves a - e) performed in 0.5 mol dm^{-3} H_2SO_4 containing CH_3OH at concentrations, (a) 0; (b) 0.5; (c) 1; (d) 3; and (e) 5 mmol dm^{-3}. Single voltammetric peaks characteristic of methanol oxidation have been developed at potentials 0.65-0.7 V. The peak potentials are slightly concentration-dependent but the voltammetric peak-responses are well-defined. The dashed line stands for the typical response of PtRu nanoparticles in the methanol-free electrolyte. It is apparent from Figure S11B (Curves a – d) that the oxidation methanol under chronoamperometric conditions is also concentration-dependent.

The proportionality of the peak-current and chronoamperometric current densities on concentration is evident from Figures 11C and 11D. The obtained here working curves have been further used for analytical determination of methanol generated during CO_2R. To perform analytical determinations, an additional working electrode modified with PtRu catalytic nanoparticles (deposited on glassy carbon at the loading 100 μg cm^{-2}) has been placed in the vicinity of the Cu/WO_3-modified large-surface-area working electrode. By comparing the net voltammetric-peak-current densities (recorded for samples after electrolysis) with the analogous current-densities values originating from blank experiments performed at different concentrations (working curves in Figure 11C and 11D), the CH_3OH-concentration generated following long-term CO_2R at Cu/WO_3 –modified electrode upon application of –0.45 V has been estimated to be equal to $5.9*10^{-4}$ mol dm^{-3}.

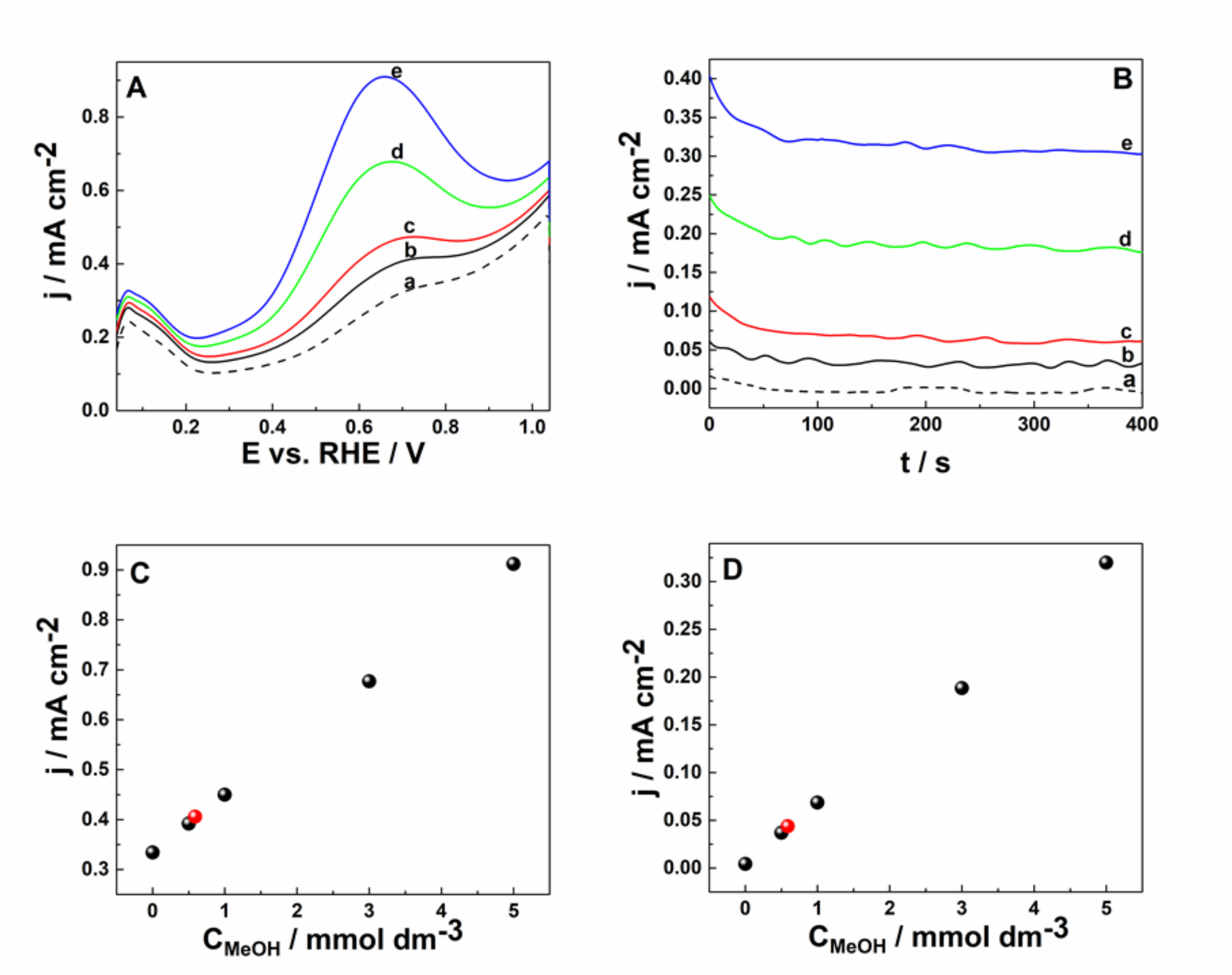

Figure S11. (**A**) Voltammetric oxidation of methanol (Curves a-e) performed at glassy carbon electrode modified with PtRu nanoparticles in deaerated 0.5 mol dm^{-3} H_2SO_4 electrolyte containing CH_3OH at concentrations, (a) 0; (b) 0.5; (c) 1; (d) 3; and (e) 5 mmol dm^{-3}. Scan rate, 10 mV s^{-1}. (**B**) Chronoamperometric responses recorded upon application of 0.54 V during oxidation of methanol. Other conditions as for Figure S11A. Working curves (**C**) and (**D**) have been prepared on the basis of voltammetric and chronoamperometric data, respectively. The point characteristic of the analyzed sample is marked in red.

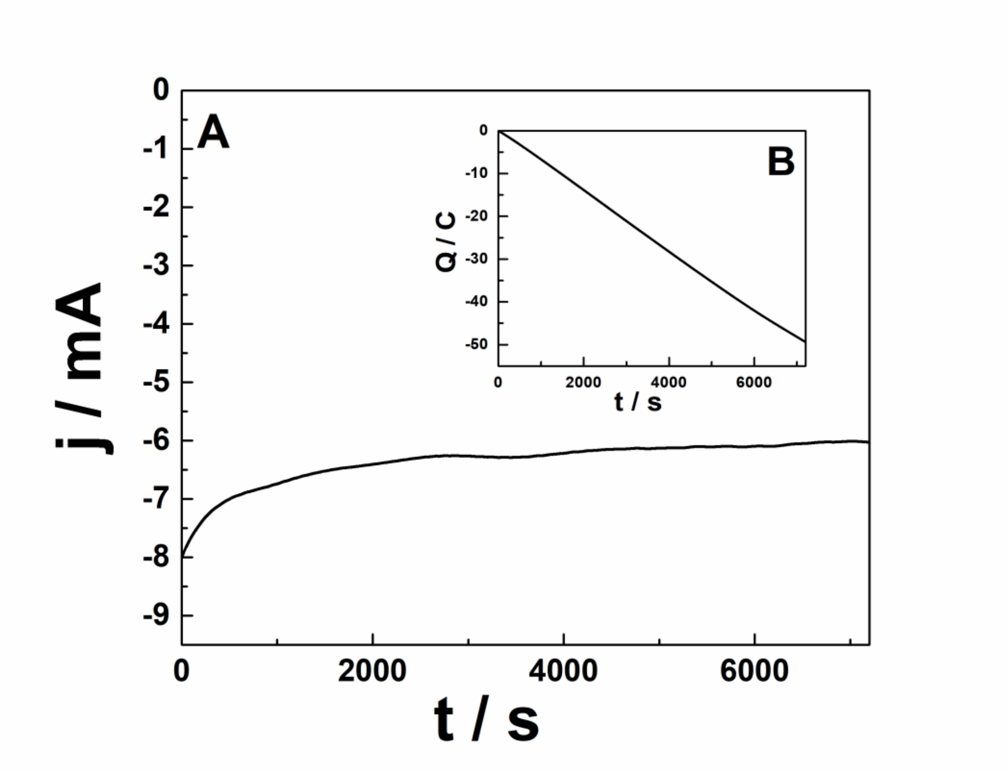

Figure S12. (**A**) Current – time dependence recorded during long-term (2h/7200s) electrolysis at Cu/WO_3-modified large-surface-area (2 cm^2) working electrode. (**B**) The dependence of charge (Q) (passed and accumulated during electrolysis) on time.
